# Evolution of Humoral and Cellular Immunity Post–Breakthrough Coronavirus Disease 2019 in Vaccinated Patients With Hematologic Malignancy Receiving Tixagevimab-Cilgavimab

**DOI:** 10.1093/ofid/ofad550

**Published:** 2023-11-02

**Authors:** Victoria G Hall, Thi H O Nguyen, Lilith F Allen, Louise C Rowntree, Lukasz Kedzierski, Brendon Y Chua, Chhay Lim, Natalie R Saunders, Emily Klimevski, Gayani S Tennakoon, John F Seymour, Vikas Wadhwa, Natalie Cain, Kim L Vo, Suellen Nicholson, Theo Karapanagiotidis, Deborah A Williamson, Karin A Thursky, Timothy Spelman, Michelle K Yong, Monica A Slavin, Katherine Kedzierska, Benjamin W Teh

**Affiliations:** Department of Infectious Diseases, Peter MacCallum Cancer Centre, Melbourne, Australia; Sir Peter MacCallum Department of Oncology, University of Melbourne, Parkville, Australia; Department of Microbiology and Immunology, University of Melbourne, Peter Doherty Institute for Infection and Immunity, Parkville, Australia; Department of Microbiology and Immunology, University of Melbourne, Peter Doherty Institute for Infection and Immunity, Parkville, Australia; Department of Microbiology and Immunology, University of Melbourne, Peter Doherty Institute for Infection and Immunity, Parkville, Australia; Department of Microbiology and Immunology, University of Melbourne, Peter Doherty Institute for Infection and Immunity, Parkville, Australia; Department of Microbiology and Immunology, University of Melbourne, Peter Doherty Institute for Infection and Immunity, Parkville, Australia; Department of Microbiology and Immunology, University of Melbourne, Peter Doherty Institute for Infection and Immunity, Parkville, Australia; Global Station for Zoonosis Control, Global Institution for Collaborative Research and Education, Hokkaido University, Sapporo, Hokkaido, Japan; Department of Infectious Diseases, Peter MacCallum Cancer Centre, Melbourne, Australia; Department of Infectious Diseases, Peter MacCallum Cancer Centre, Melbourne, Australia; Department of Infectious Diseases, Peter MacCallum Cancer Centre, Melbourne, Australia; Department of Infectious Diseases, Peter MacCallum Cancer Centre, Melbourne, Australia; Sir Peter MacCallum Department of Oncology, University of Melbourne, Parkville, Australia; Department of Hematology, Peter MacCallum Cancer Centre and Royal Melbourne Hospital, Melbourne, Australia; Department of Ambulatory Services, Peter MacCallum Cancer Centre, Melbourne, Australia; Victorian Infectious Diseases Reference Laboratory, The Royal Melbourne Hospital, Peter Doherty Institute for Infection and Immunity, Melbourne, Australia; Victorian Infectious Diseases Reference Laboratory, The Royal Melbourne Hospital, Peter Doherty Institute for Infection and Immunity, Melbourne, Australia; Victorian Infectious Diseases Reference Laboratory, The Royal Melbourne Hospital, Peter Doherty Institute for Infection and Immunity, Melbourne, Australia; Victorian Infectious Diseases Reference Laboratory, The Royal Melbourne Hospital, Peter Doherty Institute for Infection and Immunity, Melbourne, Australia; Victorian Infectious Diseases Reference Laboratory, The Royal Melbourne Hospital, Peter Doherty Institute for Infection and Immunity, Melbourne, Australia; Department of Infectious Diseases, Peter MacCallum Cancer Centre, Melbourne, Australia; Sir Peter MacCallum Department of Oncology, University of Melbourne, Parkville, Australia; Department of Biostatistics and Epidemiology, Peter MacCallum Cancer Centre, Melbourne, Australia; Centre for Population Health, Burnet Institute, Melbourne, Australia; Department of Infectious Diseases, Peter MacCallum Cancer Centre, Melbourne, Australia; Sir Peter MacCallum Department of Oncology, University of Melbourne, Parkville, Australia; Department of Infectious Diseases, Peter MacCallum Cancer Centre, Melbourne, Australia; Sir Peter MacCallum Department of Oncology, University of Melbourne, Parkville, Australia; Department of Microbiology and Immunology, University of Melbourne, Peter Doherty Institute for Infection and Immunity, Parkville, Australia; Global Station for Zoonosis Control, Global Institution for Collaborative Research and Education, Hokkaido University, Sapporo, Hokkaido, Japan; Department of Infectious Diseases, Peter MacCallum Cancer Centre, Melbourne, Australia; Sir Peter MacCallum Department of Oncology, University of Melbourne, Parkville, Australia

**Keywords:** breakthrough, COVID-19, hematologic malignancy, monoclonal antibody, vaccination

## Abstract

**Background:**

In-depth immunogenicity studies of tixagevimab-cilgavimab (T-C) are lacking, including following breakthrough coronavirus disease 2019 (COVID-19) in vaccinated patients with hematologic malignancy (HM) receiving T-C as pre-exposure prophylaxis.

**Methods:**

We performed a prospective, observational cohort study and detailed immunological analyses of 93 patients with HM who received T-C from May 2022, with and without breakthrough infection, during a follow-up period of 6 months and dominant Omicron BA.5 variant.

**Results:**

In 93 patients who received T-C, there was an increase in Omicron BA.4/5 receptor-binding domain (RBD) immunoglobulin G (IgG) antibody titers that persisted for 6 months and was equivalent to 3-dose-vaccinated uninfected healthy controls at 1 month postinjection. Omicron BA.4/5 neutralizing antibody was lower in patients receiving B-cell–depleting therapy within 12 months despite receipt of T-C. COVID-19 vaccination during T-C treatment did not incrementally improve RBD or neutralizing antibody levels. In 16 patients with predominantly mild breakthrough infection, no change in serum neutralization of Omicron BA.4/5 postinfection was detected. Activation-induced marker assay revealed an increase in CD4^+^ (but not CD8^+^) T cells post infection, comparable to previously infected healthy controls.

**Conclusions:**

Our study provides proof-of-principle for a pre-exposure prophylaxis strategy and highlights the importance of humoral and cellular immunity post–breakthrough COVID-19 in vaccinated patients with HM.

## Teaser

In 93 vaccinated patients with hematologic malignancy receiving tixagevimab-cilgavimab, humoral and cellular immunity was assessed. In 16 patients with mild breakthrough infection, there was no change in serum neutralization of Omicron BA.4/5, but there was an increase in CD4^+^ T cells postinfection.

Multiple strategies including vaccination and early antiviral therapies are now in place to reduce the risk of severe coronavirus disease 2019 (COVID-19) in patients with hematologic malignancy (HM). Despite these measures, patients with HM remain at high risk of morbidity and mortality from breakthrough (BT) infection [1, 2]. Tixagevimab-cilgavimab (T-C) offers an additional preventive strategy for high-risk patients [3]. It is a long-acting monoclonal antibody combination that binds nonoverlapping portions of the ancestral severe acute respiratory syndrome coronavirus 2 (SARS-CoV-2) spike protein receptor-binding domain (RBD), preventing viral–human angiotensin-converting enzyme 2 (ACE2) receptor interaction and cell entry [3]. In Australia, T-C was first prioritized for HM patients in April 2022 at a dose of 150/150 mg, coinciding with emergence of Omicron/BA.5 [4–6].

Clinical effectiveness studies of T-C have reported a modest BT infection rate (3%–15%) in patients with HM, depending on the prevailing variant, underlying disease, follow-up period, and COVID-19 prevalence [3, 7–10]. Immunogenicity studies of HM patients who have received T-C have had short follow-up and limited assessment of humoral immunity and lacked a healthy control group for comparison [7, 11, 12]. There has also been limited immunological evaluation of BT infection. Although T-C is no longer in clinical use due to lack of neutralizing activity to current Omicron subvariants [13], monoclonal antibodies as pre-exposure prophylaxis to respiratory viruses are under assessment for high-risk populations including for COVID-19 (AZD5156) and respiratory syncytial virus [14, 15].

To guide future monoclonal antibody preexposure prophylaxis studies, we report the dynamics of humoral and cellular immune responses in a cohort of vaccinated patients with HM receiving T-C as pre-exposure prophylaxis during Omicron BA.5 and the evolution of immune responses with BT infection in comparison to a healthy control population.

## METHODS

### Study Design and Cohorts

An observational, prospective, single-center cohort study was performed from 1 May 2022 of adult patients ≥18 years of age with HM eligible to receive T-C (150/150 mg). The primary outcome was the development of BT COVID-19 infection after T-C with planned follow-up of 6 months post injection. Breakthrough COVID-19 was defined as compatible symptoms and positive detection by polymerase chain reaction or rapid antigen testing for SARS-CoV-2, occurring any time after T-C. Sequencing of SARS-CoV-2 variants of concern was not routinely reported for individual patients, but is monitored by a national real-time genomic surveillance program [5, 6, 16]. Severity of COVID-19 was assessed as per established criteria [17].

Patients were recruited from the outpatient setting. Exclusion criteria included febrile illness in the preceding week, recipient of intravenous immunoglobulin (±30 days), and severe thrombocytopenia or other coagulopathy that precluded safe intramuscular injection of T-C.

Data including demographics, HM treatment, COVID-19 infection, and vaccination history were collected and recorded into an electronic database (REDCap, Vanderbilt University). Blood was collected from each participant for sera, plasma, granulocytes, and peripheral blood mononuclear cells (PBMCs) at a baseline visit prior to T-C (up to −30 days, V0), then days 30 (V1), 90 (V3), and 180 (V6) after injection (±2 weeks). Patients were followed closely with monthly phone calls for BT COVID-19, attributable side effects, and/or further COVID-19 vaccination. Patients were able to additionally consent to blood collection 2–4 weeks post–COVID-19 vaccination or infection.

### Healthy Controls

Healthy control participants were included from the Victorian Critical Vaccinees Collection (VC^2^), a prospectively collected, ethics-approved (HREC/75984/Austin-2021) blood sample biobank of COVID-19–vaccinated individuals. Samples from 2 control groups were utilized: (1) 15 healthy participants with no history of COVID-19 after 3 COVID-19 vaccine doses (cohort 1); and (2) 10 healthy participants after mild BT COVID-19 during Omicron BA.1 and 2-dose vaccinated (cohort 2).

### Assessment of SARS-CoV-2–Specific Antibodies

Plasma antibodies against ancestral and Omicron/BA.4/5 SARS-CoV-2 RBD protein (Acro Biosystems, Newark, Delaware) were assessed by immunoglobulin G (IgG) enzyme-linked immunosorbent assay as previously described [18, 19]. All samples were assessed for serological evidence of previous SARS-CoV-2 infection using the Roche Cobas Elecsys anti–SARS-CoV-2 nucleocapsid assay (Roche Diagnostics GmbH, Mannheim, Germany) at the baseline visit and post–BT infection, with ≥1.0 U/mL considered reactive.

### Assessment of Neutralizing Antibodies

The GenScript SARS-CoV-2 surrogate virus neutralization test (sVNT) (GenScript USA, Piscataway, New Jersey) was performed with ancestral and BA.4/5 Omicron RBD constructs (GenScript number L00847-A) as described previously [18, 20–22]. This assay works by incubating serum with horseradish peroxidase–conjugated spike RBD and then transferring the mixture to ACE2-coated wells. If neutralizing antibodies are present in the serum, then RBD–ACE2 interactions are blocked. The sVNT measures total neutralizing antibodies in sera. A positive result was defined as a neutralizing antibody threshold of ≥30% neutralization/inhibition as per the manufacturer, with reported sensitivity and specificity for the assay of 93.8% and 99.4%, respectively.

### 24-Hour Stimulation With Spike Overlapping Peptide Pools

PBMCs were thawed and plated into a 96-well plate at 1 × 10^6^ PBMCs/well. For the activation-induced marker (AIM) assay, cells were stimulated in complete RPMI with 10 mg/mL SARS-CoV-2 spike peptide pool (181 peptides, 0.06 mg/mL per peptide; BEI Resources, National Institute of Allergy and Infectious Diseases, National Institutes of Health, SARS-Related Coronavirus 2 spike glycoprotein, NR-52402) or dimethyl sulfoxide (DMSO) (0.1%; Sigma), as a negative control, and cultured at 37°C/5% CO_2_ for 24 hours. Cells were washed and stained (antibody panel, Supplementary Table 1) before fixing with 1% paraformaldehyde. Values obtained for PBMCs cultured with DMSO under the same conditions (negative controls) were subtracted from peptide-stimulated values. Activated CD4^+^ T cells were defined as CD137^+^CD134^+^ and activated CD8^+^ T cells were defined as CD137^+^CD69^+^.

Spike and non-spike epitope-specific tetramer^+^ T-cell responses as described previously [23–26] by peptide-HLA tetramer-associated magnetic enrichment (TAME) assay was performed for a subset of patients post–BT infection (Supplementary Methods).

For cell-mediated immunity analyses, samples were acquired on a LSRII Fortessa using the software BD FACSDiva version 8.0.1, and flow cytometry data were analyzed using FlowJo software version 10.

### Statistical Analysis

The primary analysis focused on comparisons between HM patients, with Mann-Whitney *U* test (unpaired) and Wilcoxon signed-rank test (paired) used for comparisons between 2 groups. Given the HM patients acted as their own controls between time points, over a relatively short period of time (6 months), these analyses were not adjusted for. Key clinically relevant questions were analyzed by subgroup analysis including the effect of vaccination post T-C, receipt of B-cell–depleting therapy within the last 12 months, and BT infection. McNemar test was used to compare proportions of positive versus negative Omicron BA.4/5 sVNT at each time point in patients with HM who received T-C.

Univariable and multivariable linear mean regression or quantile median regression were used to compare HM cases and healthy controls at baseline (V0) as appropriate. All outcome variables including IgG titers, neutralization, and T-cell frequency were formally tested for skew, with normally distributed outcome variables being compared using mean regression and significantly skewed outcomes analyzed with median regression. All outcomes were adjusted for age, sex, and time from last vaccine dose until blood sample (cohort 1) or age, sex, and time from BT infection until blood sample (cohort 2).

For all analyses, *P* < .05 was considered significant. Figures were prepared using GraphPad Prism version 9 software. All statistical analyses were performed using IBM SPSS (version 28.0), GraphPad Prism (version 9), or Stata (version 17) software.

### Patient Consent Statement

Approval (HREC/85611/PMCC) was granted from the Human Research Ethics Committee of the Peter MacCallum Cancer Centre, and patients enrolled provided written informed consent.

## RESULTS

**Figure 1.**
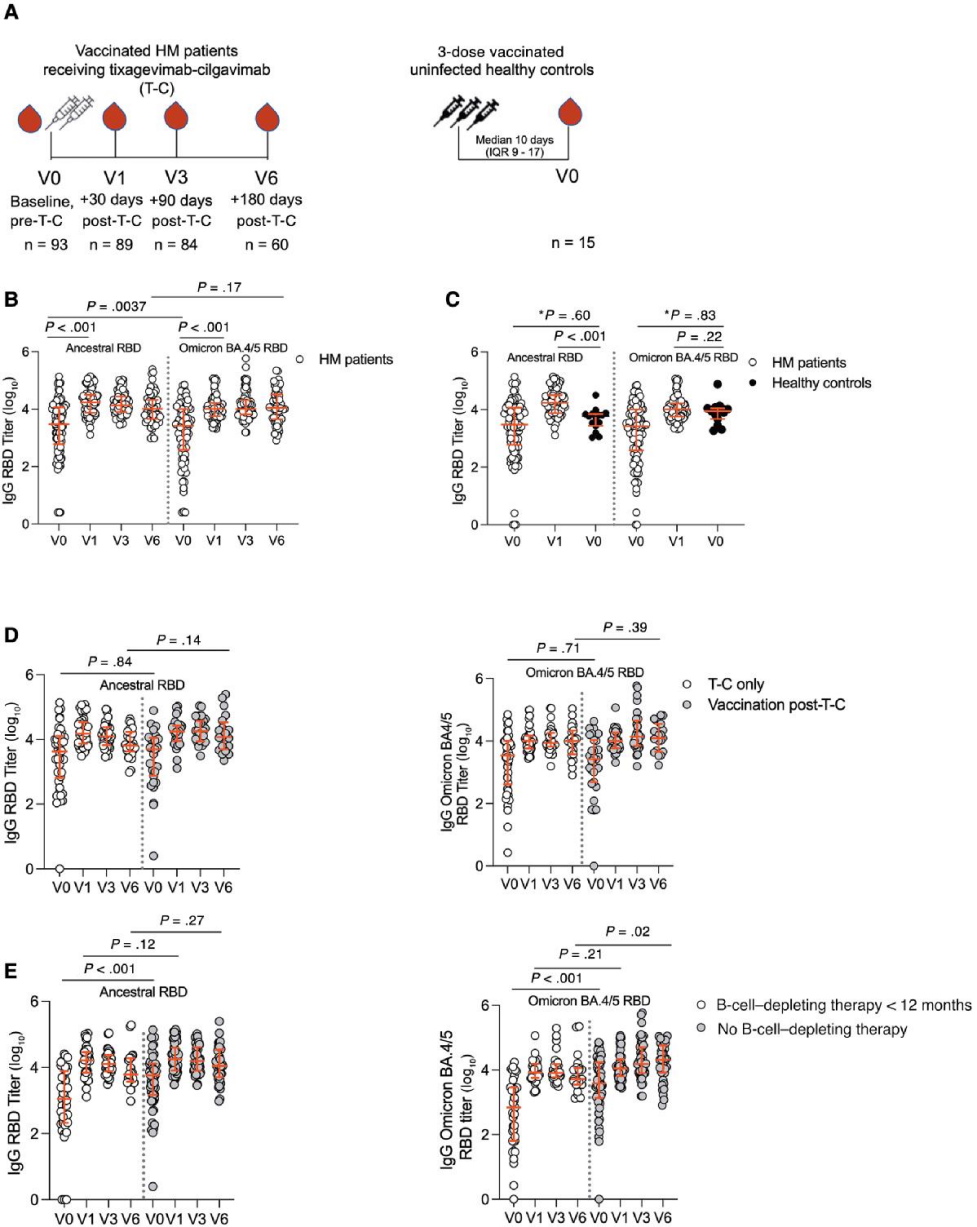
Severe acute respiratory syndrome coronavirus 2 (SARS-CoV-2) anti–receptor-binding domain (RBD) immunoglobulin G (IgG) titers in the overall cohort. *A*, Study cohort schema of patients with hematologic malignancy (HM) and 3-dose-vaccinated uninfected healthy controls. For patients with HM, blood was collected prior to tixagevimab-cilgavimab treatment (T-C; V0, n = 93) and at 1 mo (V1, n = 89), 3 mo (V3, n = 84), and 6 mo (V6, n = 60) postinjection. *B*, Ancestral and Omicron BA.4/5 SARS-CoV-2 RBD IgG titers for the entire HM patient cohort over the study period. *C*, Anti-RBD IgG titers against ancestral and Omicron BA.4/5 RBD for the entire cohort of patients with HM (n = 93) compared to 3-dose-vaccinated healthy controls without a history of coronavirus disease 2019 (COVID-19) (n = 15). *V0 timepoint analyzed by adjusted quantile median regression analysis. V0 (healthy controls) vs V1 (HM patients) timepoint comparison analyzed by unadjusted Mann-Whitney *U* test. *D*, Comparison of ancestral and Omicron BA.4/5 anti-RBD IgG titers in patients with HM who received T-C and then COVID-19 vaccination (n = 31, vaccination post-T-C) during the study period compared to those who only received T-C (n = 47, T-C only). *E*, SARS-CoV-2 ancestral and Omicron BA.4/5 anti-RBD IgG titers in patients with HM receiving B-cell–depleting therapy in the last 12 months (n = 40) compared to those who did not receive B-cell–depleting therapy (n = 53). Horizontal lines represent median with interquartile range (IQR). For unadjusted analyses, Wilcoxon matched-pairs signed-rank test was used to compare titers at different time points within the same group and Mann-Whitney *U* test to compare titers between groups.

We enrolled 93 patients with HM (Figure 1*A*) who received T-C at a dose of 150 mg/150 mg from 1 May to August 2022. Clinical characteristics of BT infection in this cohort have been described elsewhere [27]. The majority of patients (Table 1) were male (59.1%) with a median age of 62.6 years. Receipt of active HM treatment (44.1%) was the most common indication for T-C. Most had received ≥3 COVID-19 vaccine doses (80/93 [86.0%]) at a median of 111 days (interquartile range [IQR], 64–160 days) prior to T-C. After T-C, 31 of 93 (33.3%) received additional vaccination against COVID-19. Of those vaccinated after T-C (Table 1), 3 patients received the bivalent BA.1 Omicron vaccine mRNA-1273.214. All others (28/31 patients [90.3%]) received monovalent vaccination containing ancestral virus. There were 14 of 93 participants (15.1%) with prior history of COVID-19 (within 6 months), but only 6 of these (42.9%) had a reactive anti-nucleoprotein antibody at V0. Eleven patients received a second dose of T-C ≥14 days prior to V6 and were excluded from subsequent V6 analysis.

**Table 1.** Baseline Demographics of Patients With Hematologic Malignancy

Characteristic	All Participants (N = 93)
Sex, male	55 (59.1)
Age, y, median (IQR)	62.6 (55.1–69.3)
No. of comorbidities, median (IQR)	0 (0–1)
Indication for T-C and underlying disease	
Allogeneic SCT	19 (20.4)
Acute myeloid leukemia	5 (5.38)
Acute lymphoblastic leukemia	1 (1.08)
Chronic lymphocytic leukemia	1 (1.08)
Chronic myeloid leukemia	1 (1.08)
Myelofibrosis	4 (4.30)
Aplastic anemia	2 (2.15)
Sezary syndrome	2 (2.15)
T-cell prolymphocytic leukemia	1 (1.08)
Hodgkin lymphoma	1 (1.08)
Myeloma and plasma cell disorders	1 (1.08)
Autologous SCT	23 (24.7)
Myeloma and plasma cell disorders	21 (22.6)
Diffuse large B-cell lymphoma	2 (2.15)
CAR-T therapy	10 (10.8)
Diffuse large B-cell lymphoma	8 (8.60)
Mantle cell lymphoma	1 (1.08)
Myeloma and plasma cell disorders	1 (1.08)
HM on active therapy	41 (44.1)
Chronic lymphocytic leukemia	15 (16.1)
Diffuse large B-cell lymphoma	11 (11.8)
Follicular lymphoma	5 (5.38)
Acute myeloid leukemia	3 (3.23)
Myeloma and plasma cell disorders	3 (3.23)
Marginal zone lymphoma	2 (2.15)
Mantle cell lymphoma	1 (1.08)
Myelodysplastic syndrome/myeloproliferative neoplasm overlap syndrome	1 (1.08)
Time from HM diagnosis to T-C, y, median (IQR)	2.36 (0.97–5.98)
Timing of T-C	
Pre–cellular therapy (transplant or CAR-T)	8 (8.60)
Timing of T-C prior to cellular therapy, d, median (IQR)	33 (22–56)
Post–T-C cellular therapy (transplant or CAR-T)	44 (47.3)
Timing of T-C post–cellular therapy, d, median (IQR)	351 (72–918)
Receiving active therapy at time of T-C	43 (46.2)
Bruton tyrosine kinase inhibitor, current	6 (6.45)
Bispecific antibody therapy in last 12 mo	7 (7.53)
Anti-CD20 therapy in last 12 mo	35 (37.6)
Time from last chemotherapy to T-C, d, median (IQR)	34 (0–252)
No. of previous lines of therapy, median (IQR)	2 (1–2)
Underlying disease in remission	70 (75.2)
Immunosuppression at study entry	
Lymphocyte count, × 10^9^/L, median (IQR)	1.0 (0.7–1.7)
Neutrophil count, × 10^9^/L, median (IQR)	2.90 (2.1–4.3)
Hypogammaglobulinemia <4 g/L	14 (15.1)
Vaccination status	
≥3 COVID-19 vaccines prior to T-C	80 (86.0)
In those ≥3 dose vaccinated, prior to T-C, time from last COVID-19 vaccine, d, median (IQR)	111 (64–160)
Post T-C, No. of participants receiving COVID-19 vaccine	31 (33.3)
BNT162b2 (monovalent), No. (%) of those vaccinated post-T-C (n = 31)	20 (64.5)
mRNA-1273 (monovalent), No. (%) of those vaccinated post-T-C (n = 31)	7 (22.6)
Novavax, No. (%) of those vaccinated post-T-C (n = 31)	1 (3.2)
Bivalent BA.1 Omicron vaccine (mRNA-1273.214), No. (%) of those vaccinated post-T-C (n = 31)	3 (9.7)
Pre–T-C SARS-CoV-2 infection	14 (15.1)
Time from preceding SARS-CoV-2 infection to T-C, d, median (IQR)	89 (35–183)

Data are presented as No. (%) unless otherwise indicated.

Abbreviations: CAR-T, chimeric antigen receptor T cell; COVID-19, coronavirus disease 2019; HM, hematologic malignancy; IQR, interquartile range; SARS-CoV-2, severe acute respiratory syndrome coronavirus 2; SCT, stem cell transplant; T-C, tixagevimab-cilgavimab.

### IgG Titers Against SARS-CoV-2 Ancestral RBD and Omicron BA.4/5 RBD

With T-C, there was an expected rise in median IgG titers against ancestral and Omicron BA.4/5 RBD IgG (Figure 1*B*) at V1 (*P* < .001 for both). There was no difference in the overall pattern when patients with prior COVID-19 were excluded (Supplementary Figure 1*A*). We compared these responses with fifteen 3-dose-vaccinated healthy controls (Supplementary Table 2) with no history of COVID-19 (Figure 1*A*) at V0 (Supplementary Table 3) and unadjusted analysis at V1. On adjusted analysis, prior to T-C (Figure 1*C*), patients with HM had similar ancestral IgG RBD titers (*P* = .60) and Omicron BA.4/5 RBD IgG (*P* = .83). However, at V1, median ancestral IgG titers were higher than those in healthy controls (*P* < .001) with similar median IgG Omicron BA.4/5 titers (*P* = .22).

There was no difference in median IgG titers (Figure 1*D*) against ancestral or Omicron BA.4/5 RBD in patients at V0 (*P* = .84 and *P* = .71, respectively) or V6 (*P* = .14 and *P* = .39, respectively) in those who had either received T-C alone (n = 47) or had received T-C and vaccination (n = 31) during the study.

In patients who had received any B-cell–depleting therapies in the last 12 months (n = 40), median V0 IgG titers against ancestral RBD and Omicron BA.4/5 RBD (Figure 1*E*) were significantly lower than participants who had not received any B-cell–depleting therapy (both *P* < .001). In patients receiving B-cell therapy, levels were similar against the ancestral RBD (*P* = .27) but lower against the Omicron BA.4/5 RBD (*P* = .02) at V6. The effect of therapies received (Supplementary Figure 1*B*) and underlying disease type (Supplementary Figure 1*C* and 1*D*) is provided, with lower IgG values against ancestral and Omicron BA.4/5 RBD noted in patients receiving B-cell–depleting therapy compared to cellular (hematopoietic cell transplant [HCT] or chimeric antigen receptor T-cell [CAR-T] therapy) or other therapies and in patients with aggressive or indolent lymphoma, who are common recipients of B-cell–depleting therapies.

In summary, a significant rise in the ancestral and Omicron BA.4/5 RBD IgG was observed after T-C in patients with HM, achieving levels comparable to 3-dose-vaccinated uninfected healthy controls, with no additional serological benefit seen in those receiving vaccination in the 6-month follow-up period and negative influence in those who had received recent B-cell–depleting therapy.

#### Neutralization of SARS-CoV-2 Ancestral and Omicron BA.4/5 Strains

**Figure 2.**
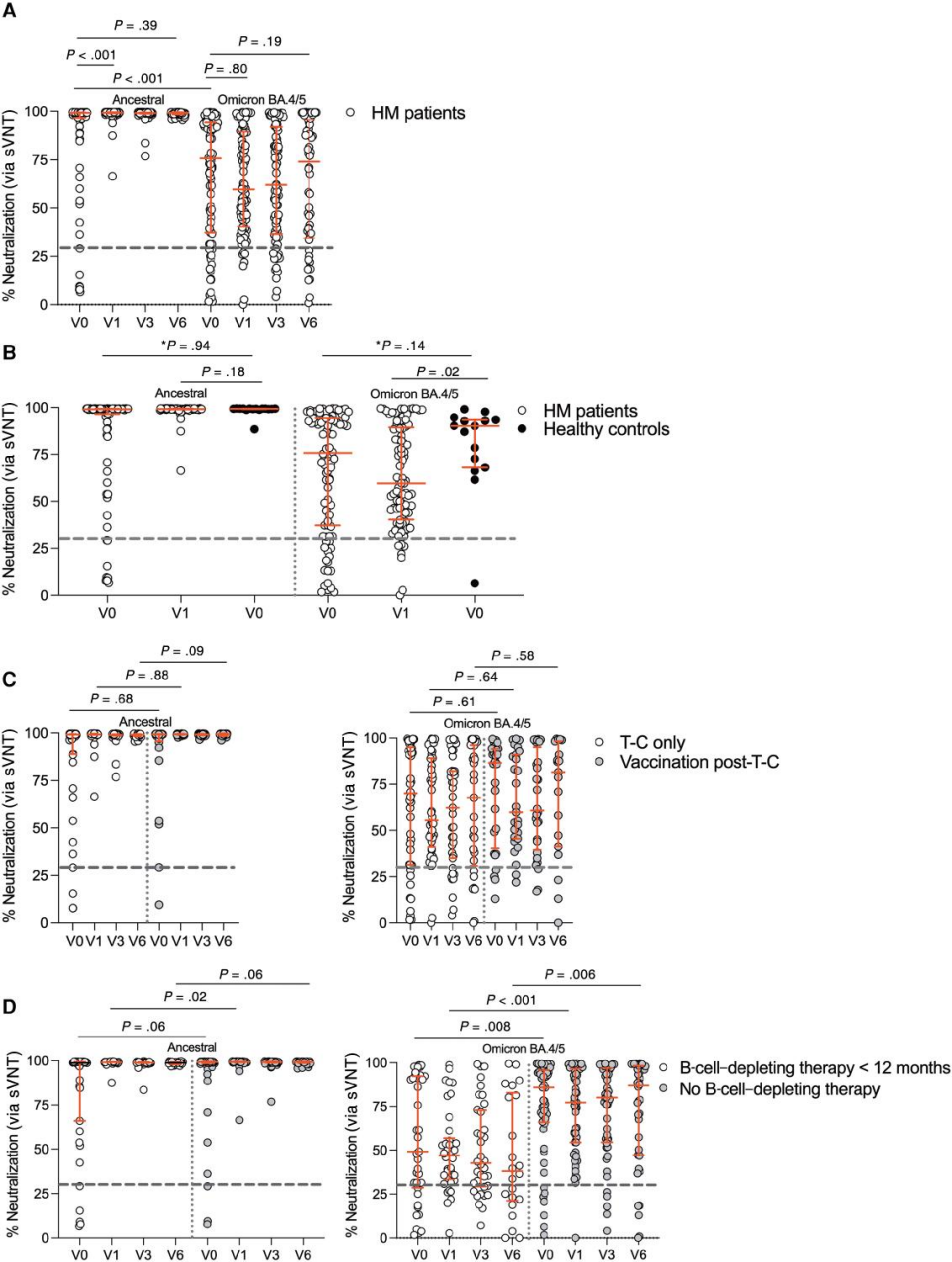
Overall cohort surrogate virus neutralization test (sVNT) against ancestral and Omicron BA.4/5 severe acute respiratory syndrome coronavirus 2 (SARS-CoV-2) strains. *A*, Percentage neutralization against ancestral and Omicron BA.4/5 RBD for the entire cohort (n = 93) over the study period, with blood collected prior to tixagevimab-cilgavimab treatment (T-C; V0, n = 93) and at 1 mo (V1, n = 89), 3 mo (V3, n = 84), and 6 mo (V6, n = 60) postinjection. *B*, Comparison of sVNT for patients with hematologic malignancy (HM) and healthy controls. Percentage neutralization against ancestral and Omicron BA.4/5 RBD for the entire cohort of patients with HM (n = 93) compared to 3-dose-vaccinated healthy controls without a history of coronavirus disease 2019 (n = 15). *V0 timepoint analyzed by adjusted quantile median regression analysis. V0 (healthy controls) vs V1 (HM patients) timepoint comparison analyzed by unadjusted Mann-Whitney *U* test. *C*, Comparison of ancestral and Omicron BA.4/5 sVNT in patients with HM who received T-C and vaccination (n = 31, vaccination post-T-C) during the study period compared to those who only received T-C (n = 47, T-C only). *D*, Ancestral and Omicron BA.4/5 sVNT in patients receiving B-cell–depleting therapy in the last 12 mo (n = 40, B-cell–depleting therapy) compared to patients with HM who did not (n = 53, none). Positive neutralization inhibition percentage of ≥30% was used as per manufacturer's instructions, represented by dotted line. Horizontal lines represent median with interquartile range. For unadjusted analyses, Wilcoxon matched-pairs signed-rank test was used to compare values at different time points within the same group and Mann-Whitney *U* test to compare values between groups.

For the overall cohort of patients with HM (Figure 2*A*), sVNT on sera against ancestral and Omicron BA.4/5 strains was performed for all time points. In contrast to the IgG titers, at V1 (Figure 2*A*), there was improvement in percentage neutralization against ancestral (*P* < .001) but not Omicron BA.4/5 (*P* = .80). The findings remained consistent when patients with prior COVID-19 were excluded from the analysis (Supplementary Figure 2*A*). The proportion of patients with HM achieving a positive sVNT ≥30% and ≥50% against ancestral and Omicron BA.4/5 strains was analyzed (Supplementary Table 4). At V1, all HM patients had positive neutralization against ancestral RBD and this persisted until V6. This is distinct to sVNT against Omicron BA.4/5, where the proportion of patients achieving ≥30% neutralization/inhibition (Supplementary Table 4 and Supplementary Figure 2*B*) increased at V1 (*P* = .052) but then declined significantly at V3 (V1 vs V3, *P* = .021). In comparison to healthy controls, ancestral sVNT (Figure 2*B*) was equivalent at V0 on adjusted median regression analysis (Supplementary Table 5) and at V1 (*P* = .18). However, on unadjusted analysis, Omicron BA.4/5 percentage neutralization was lower than in healthy controls (*P* = .02) at V1.

There was no difference in percentage neutralization against ancestral (Figure 2*C*) or Omicron BA.4/5 (Figure 2*C*) at baseline or V6 in patients who had either received T-C alone (n = 47) or had received T-C and then vaccination (n = 31). Patients who had received B-cell–depleting therapy in the last 12 months had significantly lower Omicron BA.4/5 percentage neutralization at baseline, V1, and V6 compared to patients who had not received any B-cell–depleting therapy (Figure 2*D*). The effect of therapies received (Supplementary Figure 2*C*) and underlying disease type (Supplementary Figure 2*D*) is provided, with lower neutralization values noted in patients receiving B-cell–depleting therapies and those with aggressive or indolent lymphoma.

Therefore, sVNT was improved against the ancestral but not Omicron BA.4/5 strain after T-C over the study period, without additional benefit of vaccination, and lower levels in those receiving B-cell–depleting therapy.

### Humoral Immunity Before and After Breakthrough Infection

The overall BT infection rate in patients with HM was 20.4% (19/93) and occurred at a median of 81 days (IQR, 21–143 days) after T-C (Supplementary Table 6). Most patients had mild COVID-19 (17/19 [89.5%]); of these, 15 of 17 (88.2%) received early outpatient antiviral therapy. Three patients had COVID-19 both prior to, and after, T-C. Two patients had moderate COVID-19 requiring hospital admission, antiviral therapy, corticosteroid therapy, and low-flow oxygen therapy. No patient required intensive care, and there was no COVID-19–related mortality.

**Figure 3.**
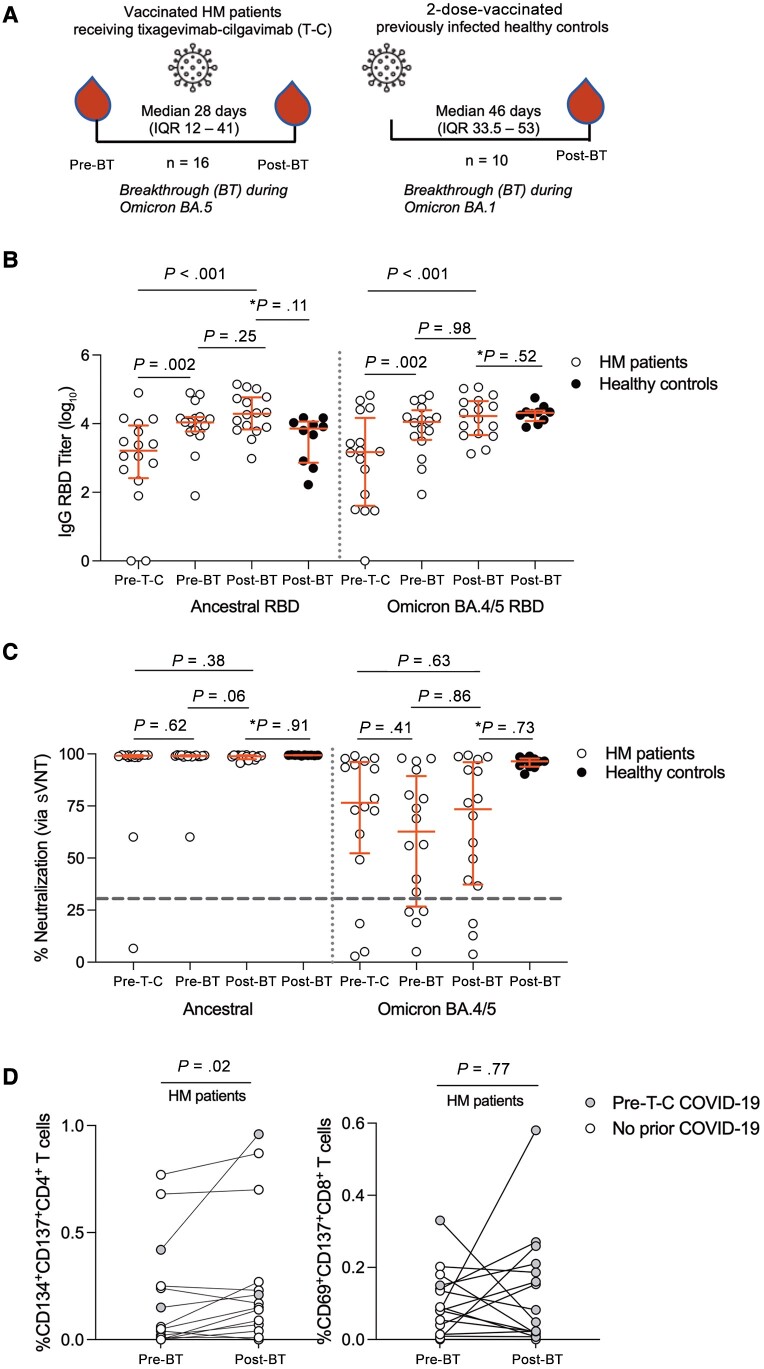
Humoral and cell-mediated immunity pre– and post–breakthrough (BT) coronavirus disease 2019 (COVID-19). *A*, Study schema of BT COVID-19 cohort for patients with hematologic malignancy (HM) and healthy controls. *B*, Plasma immunoglobulin G (IgG) receptor-binding domain (RBD) ancestral and Omicron BA.4/5 antibody titers pre– and post–BT infection. Pre–tixagevimab-cilgavimab (T-C) antibody titers also shown for comparison. HM patients, n = 16; healthy controls, n = 10. Horizontal lines represent median with interquartile range (IQR). *Post-BT timepoint (HM patients vs healthy controls) analyzed by adjusted linear mean regression analysis. *C*, Surrogate virus neutralization test (sVNT) to ancestral and Omicron BA.4/5 RBD pre– and post–BT infection. Positive neutralization inhibition of ≥30% was used per manufacturer's instructions, represented by dotted line. *Post-BT timepoint (HM patients vs healthy controls) analyzed by adjusted quantile median regression analysis. *D*, Activation-induced marker frequency of CD134^+^CD137^+^CD4^+^ T cells (left) and CD69^+^CD137^+^CD8^+^ cells (right) pre– and post–BT infection in patients with HM. For unadjusted analyses, Wilcoxon matched-pairs signed-rank test was used to compare values at different time points within the same group and Mann-Whitney *U* test to compare values between groups.

Sixteen patients with confirmed BT COVID-19 had pre- and postinfection samples available for analysis (Figure 3*A* and Supplementary Figure 3*A* and 3*B*). Blood sampling (Figure 3*A*) occurred postdiagnosis of COVID-19 at a median of 28 days (IQR, 12–41 days). On analysis of postinfection sera, only 4 of 16 (25%) patients had a reactive anti-nucleoprotein antibody. In comparison with 2-dose-vaccinated healthy controls with mild BT COVID-19 (Figure 3*A* and Supplementary Table 6), on adjusted linear mean regression (Supplementary Table 7), HM patients had similar ancestral IgG RBD titers (*P* = .11) and Omicron BA.4/5 IgG RBD titers (*P* = .52) post–BT infection (Figure 3*B*).

In patients with HM, median ancestral and Omicron BA.4/5 RBD IgG titers were significantly raised post–BT infection (Figure 3*B*) compared to pre–T-C levels (both *P* < .001) but did not change post–BT infection compared to post–T-C. This may be due to a lack of endogenous antibody production post–BT infection and/or because the median antibody level is already increased with receipt of T-C, and so no significant change was observed.

There was also no increase in percentage neutralization (Figure 3*C*) pre- and postinfection of ancestral (*P* = .06) or Omicron BA.4/5 (*P* = .86) sVNT. Individual change over these time points for IgG RBD titers and sVNT are demonstrated (Supplementary Figure 4*A* and 4*B*). In HM patients, Omicron BA.4/5 IgG RBD titers significantly correlated (Supplementary Figure 4*C*) with percentage neutralization of Omicron BA.4/5 (*r* = 0.85, *P* < .001). The proportion of patients with HM with a positive sVNT ≥30% to Omicron BA.4/5 remained comparable (13/16 [81.3%]) pre- and postinfection (Supplementary Table 8).

On adjusted median regression analysis (Supplementary Table 9), patients with HM had similar neutralization capacity of ancestral (*P* = .91) and Omicron BA.4/5 strains (*P* = .73), compared with healthy controls post–BT infection (Figure 3*C*).

### Cell-Mediated Immunity Before and After Breakthrough Infection

Representative FACS plots for CD4^+^ and CD8^+^ T cells are presented for spike-specific T-cell responses (Supplementary Figure 5*A*). Patients with HM had a significant rise (*P* = .02) in the frequency of spike-specific activated CD134^+^CD137^+^CD4^+^ T cells post–BT infection (Figure 3*D*) but not spike-specific activated CD69^+^CD137^+^CD8^+^ T cells (*P* = .77; Figure 3*D*). There was no significant influence of prior B-cell–depleting therapy in the pre- and post–BT infection frequency of activated CD4^+^ or CD8^+^ T cells (Supplementary Figure 5*B* and 5*C*).

**Figure 4.**
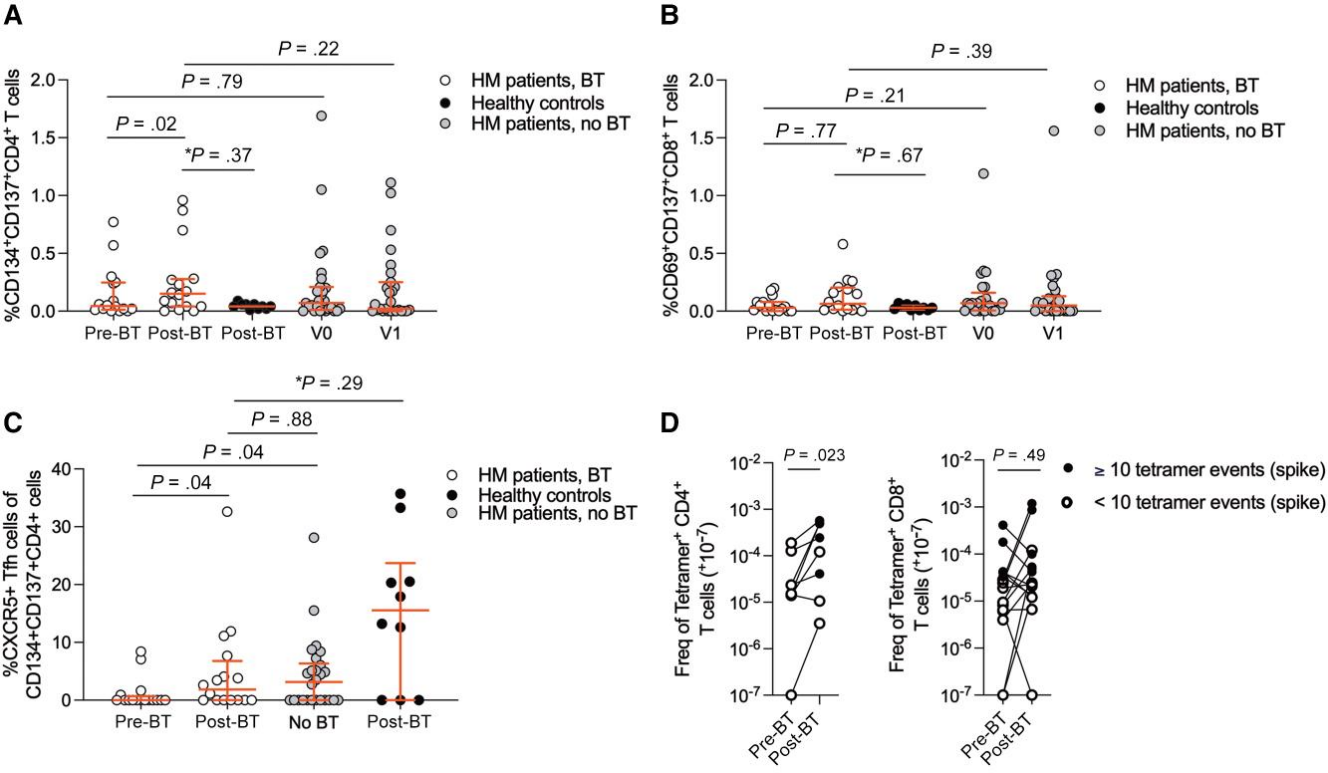
Cell-mediated immunity pre– and post–breakthrough (BT) coronavirus disease 2019 (COVID-19) in patients with hematologic malignancy (HM) and healthy controls. *A*, Activation-induced marker (AIM) frequency of CD134^+^CD137^+^CD4^+^ T cells post–BT infection in patients with HM compared to previously infected 2-dose-vaccinated healthy controls and 3-dose-vaccinated patients with HM without BT infection. *Post-BT timepoint (HM patients vs healthy controls) analyzed by adjusted quantile median regression analysis. *B*, AIM frequency of CD69^+^CD137^+^CD8^+^ T cells post–BT infection in patients with HM compared to previously infected 2-dose-vaccinated healthy controls and 3-dose-vaccinated patients with HM without BT infection. *Post-BT timepoint (HM patients vs healthy controls) analyzed by adjusted quantile median regression analysis. *C*, Percentage activated T-follicular helper cells (Tfh) pre– and post–BT infection in patients with HM, comparing vaccinated patients with HM and no BT infection (no infection) as well as 2-dose-vaccinated healthy controls postinfection. Patients with prior COVID-19 (ie, preceding study commencement) were excluded from the no infection group. *Post-BT timepoint (HM patients vs healthy controls) analyzed by adjusted quantile median regression analysis. *D*, Spike-specific tetramer^+^ CD4^+^ (left) and CD8^+^ (right) T-cell frequencies of patients with HM pre– and post–BT infection (n = 10). Frequency of tetramer^+^ T cells have been right-shifted by 10^−7^ (ie, no detected tetramer^+^ events displayed as 10^−7^) to allow for visibility on the logarithmic y-axis. Any samples with <10 tetramer^+^ events are shown as open symbols. For unadjusted analyses, Wilcoxon matched-pairs signed-rank test was used to compare values at different time points within the same group and Mann-Whitney *U* test to compare values between groups.

On adjusted median regression analysis (Supplementary Table 10), in comparison to healthy controls (Figure 4*A* and 4*B*), there was similar frequency of activated CD4^+^ T cells (*P* = .37) and CD8^+^ T cells (*P* = .67) post–BT infection in patients with HM. Healthy controls (Figure 4*C*) also had similar frequency of activated T-follicular helper cells (Tfh) (*P* = .29) in comparison to patients with HM post–BT infection, despite a rise in the percentage of activated Tfh in patients with HM (*P* = .04). Comparing HM patients with BT infection to those without a history of COVID-19 or BT infection (n = 27), there was no difference in the frequency of activated CD4^+^ T cells and CD8^+^ T cells (Figure 4*A* and 4*B*, respectively) at V1. There was also no difference in baseline frequency of activated CD4^+^ T cells and CD8^+^ T cells (Supplementary Figure 5*D* and 5*E*, respectively) in patients who went on to have BT infection compared with those who did not. Anti-RBD IgG titers and sVNT against Omicron BA.4/5 were not found to correlate with AIM frequency of CD4^+^ and CD8^+^ T cells post–BT infection in HM patients (Supplementary Figure 5*F* and 5*G*).

Consistent with AIM assay results, there was a significant increase in spike-specific CD4^+^ tetramer^+^ T cells but not spike-specific CD8^+^ tetramer^+^ T cells in 10 patients with BT infection by TAME assay (Figure 4*D*). The representative AIM and TAME assay gating strategies are provided in Supplementary Figures 6 and 7, respectively.

In summary, post–BT infection in vaccinated patients with HM, there was a significant CD4^+^ T-cell and Tfh rise but not CD8^+^ T-cell rise, with no impact on prior receipt of B-cell–depleting therapy in the last 12 months. Our data show that vaccinated patients with HM who have received T-C exhibit robust epitope-specific T-cell responses post–BT infection.

## DISCUSSION

We highlight a modest rate of BT COVID-19 (20.4%) in a highly vaccinated cohort of patients with HM receiving T-C as preexposure prophylaxis during Omicron BA.5. This higher BT infection rate compared with earlier reports [3, 5, 8, 13, 28] may be due to the increased immune evasiveness of Omicron BA.5, longer follow-up, and high community prevalence of COVID-19. Our main findings include (1) a significant rise in IgG titers against ancestral and Omicron BA.4/5 RBD in patients with HM receiving T-C comparable to healthy vaccinated controls, at levels of which were maintained for 6 months and with BT infection; (2) no significant change in neutralization ability against Omicron BA.4/5 after T-C and BT infection; (3) lack of incremental benefit of COVID-19 vaccination during the 6-month period following T-C; (4) lower Omicron BA.4/5 neutralization in patients receiving B-cell–depleting therapy despite receipt of T-C; and (5) increased frequency of activated and spike-specific CD4^+^ T cells in patients with HM after BT infection, similar to healthy controls, but no change in the frequency of activated or spike-specific CD8^+^ T cells post–BT infection.

Although Omicron BA.4 and BA.5 recombinant lineages (eg, BA.4.6 and BQ.1.1, respectively) had started to emerge in Australia toward the end of the study period (November–December 2022), Omicron BA.5 was still the predominant sublineage being sequenced (39.4% of sequences of BA.5 compared with 14.0% recombinant lineages in the reporting period 21 November–18 December 2022) [5, 6]. Prior in vitro findings suggest a considerable fold reduction in the neutralization ability of T-C against Omicron BA.5 (by a factor of 30.7) and >1000-fold reduction in neutralization against the later recombinant lineages compared to the ancestral strain [13, 29]. This is further supported by our findings of a lack of improvement in neutralization ability against Omicron BA.4/5 after receipt of T-C in HM patients. Therefore, it is not unexpected that we observed frequent BT infection in our cohort during the circulation of Omicron BA.5 and possibly later recombinant lineages, representing reduced clinical efficacy of T-C underpinned by decreased in vitro and in vivo neutralization ability compared to the ancestral strain.

The increase in IgG titers to ancestral and Omicron BA.4/5 RBD with T-C and BT infection in patients with HM, but not Omicron BA.4/5 neutralization, may represent a rise in nonneutralizing antibodies. This is critical as neutralization has been put forward as an immune correlate of protection and underscores the importance of not assessing anti-RBD IgG titers in isolation [30]. On unadjusted analysis, median ancestral virus IgG RBD titers were higher at V1 in vaccinated patients with HM at 1 month after T-C than 3-dose-vaccinated uninfected healthy controls and equivalent against Omicron BA.4/5 RBD with similar baseline values (ie, before T-C). Therefore, the administration of T-C may provide a top-up to preexisting immunity in patients with HM, rendering the median IgG levels higher against the ancestral RBD (the primary target of T-C) and equivalent against the Omicron BA.4/5 RBD compared with healthy controls. Interpretation of this finding is cautioned given it is an unadjusted analysis.

Of concern is the consistently lower anti-RBD IgG and neutralization values against Omicron BA.4/5 in patients who had received B-cell–depleting therapy within 12 months, in comparison to other therapies. This likely reflects a lack of reserve in the SARS-CoV-2–specific B-cell responses induced by their prior vaccination, which cannot be fully compensated by the provision of T-C [31, 32]. This highlights the ongoing vulnerability of this group to SARS-CoV-2 infection and the need to prioritize them for other prevention strategies. There was also no incremental benefit seen in SARS-CoV-2–specific IgG titers or neutralization capacity in patients who received COVID-19 vaccination after T-C in the study period. This helps to guide timing of COVID-19 vaccination in the setting of monoclonal therapies for optimal response. T-C around the time of cellular therapy (HCT or CAR-T therapy) may provide some protection from infection until revaccination can commence at 3–6 months postprocedure [33].

The lack of improvement in neutralization of Omicron BA.4/5 post–BT infection during circulation of Omicron BA.5 in patients with HM indicates an inadequate immune response to an autologous infection. This could also imply that a history of SARS-CoV-2 infection in vaccinated patients with HM may not confer protection to future infection, as is described in the general population [34–36]. This may relate to reduced activation of Tfh cells post–BT infection, and reflect defects in interactions between Tfh and B cells in patients with HM that are otherwise essential for the induction of effective SARS-CoV-2–specific neutralizing antibody responses [37]. Potential contributors to these outcomes include the patients’ underlying HM and/or the anticancer therapy received.

The lack of increased CD8^+^ T-cell activation may relate to the large majority of BT COVID-19 in our study being mild and/or the timing of blood sampling post–BT infection, given that CD8^+^ T cells are usually only activated for a few weeks. In immunocompetent individuals, the breadth and magnitude of T-cell responses were significantly higher in severe compared with mild cases [38]. Bange et al found that despite defects in CD4^+^ T cells, B cells, and humoral immunity, CD8^+^ T-cell responses were preserved, along with high activation of CD8^+^ T cells in patients with HM post–COVID-19 [37]. Furthermore, in patients treated with anti-CD20 therapy, a higher number of CD8^+^ T cells was associated with lower mortality and viral load [37]. Their cohort of HM patients was enriched with patients with severe COVID-19 (44.4%), sampled prior to the Omicron variant as well as the availability of COVID-19 vaccination and specific therapies [37]. The authors posited that the observed CD8^+^ T-cell response may be a compensatory reaction to a blunted humoral response [37]. This is in contrast to our cohort with BT infection, who were at least 3-dose-vaccinated and in receipt of T-C, most exhibited neutralization activity to Omicron BA.4/5 (75%) prior to infection and had an increase in Omicron BA.4/5 RBD-specific IgG response postinfection. It may be that a compensatory rise in CD8^+^ T-cell activation was not required due to humoral immunity augmented by T-C as well as virologic control being supplemented by early antiviral therapy [39]. The significant rise in spike-specific activated CD4^+^ T cells found post–BT infection in patients with HM in our cohort may represent an effort to coordinate the adaptive immune response and could be partly responsible for mild disease as previously described [40–42]. Last, there was no difference in the percentage of activated CD4^+^ and CD8^+^ T cells in HM patients with BT infection and those without infection. This agrees with findings from Nguyen et al and is reflective of a preserved T-cell response in a highly vaccinated cohort [42].

Our study does have some limitations. We employed convenience sampling for the healthy controls based on available biobank samples. Healthy controls were younger and female predominant, and the sampling time postvaccination or post–BT infection varied compared to the HM patients. To account for these potentially confounding factors we performed adjusted regression analyses at the V0 timepoint. These factors should be considered when interpreting the comparative analyses between the V0 timepoint for the healthy controls and V1 timepoint for the HM patients. Although our study examined neutralization against Omicron BA.4/5, the predominant variant during the study period, we did not examine for neutralization ability against the latest Omicron subvariants. Given our findings and recently published clinical and in vitro data, we would expect the neutralization values to be lower than what we have reported [11, 13, 43]. Due to lack of the appropriate assay availability, we could not differentiate between exogenous and endogenous antibody levels after T-C or account for the Fc portion of T-C that may contribute to immunity. We did not perform live virus neutralization testing, considered the gold standard; however, we have previously demonstrated that the sVNT correlates well [42]. As with other clinical studies, we acknowledge the limitation of a lack of comparator group in patients with HM who did not receive T-C to contrast BT infection rate and severity.

We provide a comprehensive and novel description of the dynamics of humoral and cell-mediated immune responses including for BT COVID-19 infection in patients with HM receiving T-C as preexposure prophylaxis. We identify priority groups, including patients receiving of B-cell–depleting therapy who may benefit from additional strategies, and absence of incremental immunological benefit of vaccination in the setting of T-C. This study provides proof-of-principle for this new prevention strategy and informs future use of monoclonal antibodies in development for this high-risk patient population.

## Supplementary Material

ofad550_Supplementary_Data

## Supplementary Data

Supplementary materials are available at *Open Forum Infectious Diseases* online. Consisting of data provided by the authors to benefit the reader, the posted materials are not copyedited and are the sole responsibility of the authors, so questions or comments should be addressed to the corresponding author.

## Notes

***Acknowledgments.*** This research has been conducted using samples from the Victorian Critical Vaccinees Collection (VC^2^). We acknowledge VC^2^ investigators and sites and the support of the Victorian government, and thank all participants involved. We thank BEI Resources, National Institute of Allergy and Infectious Diseases, National Institutes of Health, for providing the peptide array, SARS-related coronavirus 2 spike glycoprotein, NR-52402.

***Author contributions.*** Study design: V. G. H., B. W. T., M. A. S.., M. K. Y., K. K., T. H. O. N., J. F. S., V. W., L. F. A., and L. C. R. Data analysis: V. G. H., T. H. O. N., L. F. A., L. C. R., K. K., B. W. T., and T. S. Data collection: V. G. H., C. L., L. F. A., T. H. O. N., L. K., T. K., S. N., N. C., K. L. V., D. A. W., and L. C. R. Manuscript writing and decision to submit the manuscript for publication: All authors.

***Data availability.*** Deidentified data that support the findings of this study are available from the corresponding author upon reasonable request.

***Financial support.*** This project has received funding from a VC^2^ Research Grant (V. G. H.). V. G. H. is supported by a National Health and Medical Research Council (NHMRC) postgraduate PhD scholarship (award number 2014210). D. A. W. is funded by an NHMRC Investigator Grant (grant number APP1174555). B. W. T. is supported by the Australian government Medical Research Future Fund (MRFF) Investigator Fellowship (EL-2, award number 1195894). This work was supported by the NHMRC Leadership Investigator Grant to K. K. (grant number 1173871); an NHMRC Emerging Leadership Level 1 Investigator Grant to T. H. O. N. (grant number 1194036); and MRFF Award (award number 2016062) to K. K., T. H. O. N., L. C. R., and B. W. T.
