# Supplementary material for: Evolution of Humoral and Cellular Immunity Post–Breakthrough Coronavirus Disease 2019 in Vaccinated Patients With Hematologic Malignancy Receiving Tixagevimab-Cilgavimab
Source: Open Forum Infect Dis. 2023 Nov 2;10(11):ofad550. doi: 10.1093/ofid/ofad550 (PMC10644824; doi:10.1093/ofid/ofad550)
Supplement: ofad550_Supplementary_Data [file ofad550_supplementary_data.zip › Supplementary_Methods.docx]

**Supplementary Methods.**

*Spike and non-spike epitope-specific tetramer^+^ T cell responses*

HLA class I tetramers HLA-A*01:01/S_865_ (LTDEMIAQY), HLA-A*01:01/ORF1a_1637_ (TTDPSFLGRY), HLA-A*02:01/S_269_ (YLQPRTFLL), HLA-A*03:01/S_378_ (KCYGVSPTK), HLA-A*03:01/N_361_ (KTFPPTEPK) ,HLA-A*24:02/S_1208_ (QYIKWPWYI), HLA-B*07:02/N_105_ (SPRWYFYYL), HLA-B*15:01/S_919_ (NQKLIANQF) and HLA-B*35:01/S_321_ (QPTESIVRF) as generated by Rossjohn laboratory and validated as previously described were utilised. HLA class II tetramer HLA-DPA1*01:03/DPB1*04:01/S_167_ (TFEYVSQPFLMDLE) as generated by Rossjohn laboratory and validated as previously described were utilised.

Cryopreserved PBMCs (2-10x10^6^) underwent tetramer-associated magnetic enrichment (TAME) following staining with a class I and/or DP4 class II Spike tetramer on PE and/or another class I tetramer on APC as described. Class I and class II tetramers on PE were exclusively stained on CD8^+^ or CD4^+^ T cells, respectively, with minimal to zero non-specific binding. Using the software BD FACS DIVA v8.0.1, samples were acquired on a LSRII Fortessa and flow cytometry data were analyzed using FlowJo v10 software.
