# Supplementary material for: Evolution of Humoral and Cellular Immunity Post–Breakthrough Coronavirus Disease 2019 in Vaccinated Patients With Hematologic Malignancy Receiving Tixagevimab-Cilgavimab
Source: Open Forum Infect Dis. 2023 Nov 2;10(11):ofad550. doi: 10.1093/ofid/ofad550 (PMC10644824; doi:10.1093/ofid/ofad550)
Supplement: ofad550_Supplementary_Data [file ofad550_supplementary_data.zip › Supplementary_Tables_clean.docx]

**Supplementary Table 1. Antibody staining panel for Activation-Induced Marker assay**

| - CXCR5-BV421 (562747; BD Biosciences) - CD3-BV510 (317332; BioLegend) - CD8-BV605 (564116; BD Biosciences) - CD4-BV650 (563875; BD Biosciences) - CD25-BV711 (563159; BD Biosciences) - CXCR3-BV786 (353738; BD Biosciences) - CD137-APC (309810; BioLegend) - CD27-AF700 (560611; BD Biosciences) - CD14/CD19-APC-H7 (560180/560252; BD Biosciences) - Live/Dead NIR (L34976; Invitrogen) - CD69-PerCPCy5.5 (310925; BioLegend) - CD134-PE (340420; BD Biosciences) - CD95-PE-CF594 (562395; BD Biosciences) - CD45RA-PeCy7 (337167; BD Biosciences) |
| --- |

**Supplementary Table 2. Baseline demographics of healthy control participants without history of COVID-19 infection**

| **Characteristic** | **Healthy controls (n = 15)** |
| --- | --- |
| Age, years, median (IQR) | 34 (27.5 – 46) |
| Sex, male, n, % | 2 (13.3) |
| 3 COVID-19 vaccines prior to blood sampling, n, % | 15 (100) |
| Blood sampling time post 3^rd^ dose of vaccine, days, median, (IQR) | 10 (9 - 17.0) |
| **Co-morbidities** |  |
| Asthma | 2 (13.3) |
| Type 2 diabetes mellitus | 1 (6.7) |
| Immunosuppression | 0 |

**Supplementary Table 3. Quantile median regression analysis of ancestral and omicron BA.4/5 RBD IgG at V0 for HM patients compared with healthy controls (Figure 1C).**

| **Outcome = Figure 1C Ancestral RBD IgG V0** |  | **Median regression** | |
| --- | --- | --- | --- |
| **Factor** | **Category** | **Unadjusted β-coeficient (95% CI) p-value** | **Adjusted β-coeficient (95% CI) p-value** |
| Group | HM | -0.30 (-0.92, 0.33) 0.349 | 0.21 (-0.58, 0.99) 0.602 |
|  | Controls | Reference | Reference |
| Age (years) |  | -0.01 (-0.02, 0.01) 0.203 | -0.01 (-0.03, 0.01) 0.233 |
| Sex | Male | Reference | Reference |
|  | Female | -0.13 (-0.55, 0.28) 0.533 | -0.21 (-0.62, 0.20) 0.313 |
| Months from last dose of vaccine until V0 (baseline blood sample) |  | **-0.08 (-0.15, -0.00) 0.046** | -0.07 (-0.16, 0.01) 0.080 |
|  |  |  |  |
|  |  |  |  |
| **Outcome = Figure 1C Omicron BA.4/5 RBD IgG V0** | | **Median regression** | |
| **Factor** | **Category** | **Unadjusted β-coeficient (95% CI) p-value** | **Adjusted β-coeficient (95% CI) p-value** |
| Group | HM | -0.52 (-1.18, 0.13) 0.117 | 0.08 (-0.70, 0.87) 0.831 |
|  | Controls | Reference | Reference |
| Age (years) |  | **-0.02 (-0.03, -0.00) 0.030** | -0.01 (-0.03, 0.00) 0.158 |
| Sex | Male | Reference | Reference |
|  | Female | -0.00 (-0.44, 0.44) 0.993 | 0.01 (-0.40, 0.42) 0.974 |
| Months from last dose of vaccine until V0 (baseline blood sample) |  | **-0.11 (-0.19, -0.03) 0.008** | **-0.09 (-0.17, -0.01) 0.031** |

| **Tests of normality (p<0.05 = significant skew, median regression analysis selected)** |  |  |  |  |  |
| --- | --- | --- | --- | --- | --- |
|  |  |  |  |  |  |
| Variable | Obs | W | V | z | Prob>z |
| Figure1C Ancestral | 108 | 0.91123 | 7.817 | 4.581 | 0.00000 |
| Figure1C Omicron BA.4/5 | 108 | 0.92564 | 6.548 | 4.186 | 0.00001 |

**Supplementary Table 4. Results of sVNT against wild type and Omicron BA.4/5 at each time point for the overall cohort of patients with HM.**

|  | **Wild type** | | | | **Omicron BA.4/5** | | | |
| --- | --- | --- | --- | --- | --- | --- | --- | --- |
|  | V0 | V1 | V3 | V6 | V0 | V1 | V3 | V6 |
| **Total samples** | 93 | 89 | 84 | 60 | 93 | 89 | 84 | 60 |
| Positive SVNT ≥ 50%, n, % | 83 (89.2) | 89 (100) | 84 (100) | 60 (100) | 63 (67.7) | 56 (62.9) | 54 (64.3) | 36  (60) |
| Positive SVNT ≥ 30%, n, % | 85 (91.4) | 89 (100) | 84 (100) | 60 (100) | 76 (81.7) | 82 (92.1) | 69 (82.1) | 46 (76.7) |
| Negative SVNT < 30%, n, % | 8 (8.6) | 0 | 0 | 0 | 17 (18.3) | 7 (7.87) | 15 (17.9) | 14 (23.3) |

**Supplementary Table 5. Quantile median regression analysis of ancestral and omicron BA.4/5 sVNT at V0 for HM patients compared with healthy controls (Figure 2B).**

| **Outcome = Figure 2B Ancestral SVNT V0** |  | **Median regression** | |
| --- | --- | --- | --- |
| **Factor** | **Category** | **Unadjusted β-coeficient (95% CI) p-value** | **Adjusted β-coeficient (95% CI) p-value** |
| Group | HM | -0.25 (-0.87, 0.37) 0.427 | 0.13 (-3.24, 3.51) 0.939 |
|  | Controls | Reference | Reference |
| Age (years) |  | -0.01 (-0.03, 0.01) 0.359 | -0.01 (-0.08, 0.07) 0.889 |
| Sex | Male | Reference | Reference |
|  | Female | 0.30 (-0.35, 0.95) 0.361 | 0.29 (-1.48, 2.06) 0.748 |
| Months from last dose of vaccine until V0 (baseline blood sample) |  | -0.06 (-0.38, 0.25) 0.691 | -0.06 (-0.41, 0.30) 0.737 |
|  |  |  |  |
|  |  |  |  |
| **Outcome = Figure 2B Omicron BA.4/5 SVNT V0** | | **Median regression** | |
| **Factor** | **Category** | **Unadjusted β-coeficient (95% CI) p-value** | **Adjusted β-coeficient (95% CI) p-value** |
| Group | HM | -14.54 (-42.98, 13.90) 0.313 | 25.38 (-8.81, 59.57) 0.144 |
|  | Controls | Reference | Reference |
| Age (years) |  | -0.46 (-1.12, 0.19) 0.161 | -0.48 (-1.22, 0.26) 0.201 |
| Sex | Male | Reference | Reference |
|  | Female | 17.68 (-1.53, 36.89) 0.071 | 9.51 (-8.40, 27.43) 0.295 |
| Months from last dose of vaccine until V0 (baseline blood sample) |  | -3.03 (-6.15, 0.08) 0.056 | **-4.49 (-8.11, -0.87) 0.016** |

| **Tests of normality (p<0.05 = significant skew, median regression analysis selected)** |  |  |  |  |  |
| --- | --- | --- | --- | --- | --- |
|  |  |  |  |  |  |
| Variable | Obs | W | V | z | Prob>z (test of normality) |
| Figure2B Ancestral | 108 | 0.49025 | 44.887 | 8.475 | 0.00000 |
| Figure2B Omicron BA.4/5 | 108 | 0.84917 | 13.282 | 5.762 | 0.00000 |

**Supplementary Table 6. Characteristics of the breakthrough COVID-19 cohort included in blood sample analysis (patients with HM and healthy controls)**

| **Characteristic** | **Patients with HM (n = 16)** |
| --- | --- |
| Sex, male, n, % | 8 (50) |
| Age, years, IQR | 63.8 (58.3 – 69.0) |
| Number of co-morbidities, median, (IQR) | 1 (0-1) |
| **Indication for Tixagevimab-cilgavimab and underlying disease** |  |
| **Allogeneic SCT < 24 months, n, %** | 2 (12.5) |
| Chronic myeloid leukemia, n, % | 1 (6.25) |
| AML, n, % | 1 (6.25) |
| **Autologous SCT < 24 months, n, %** | 4 (25) |
| Diffuse large B cell lymphoma (DLBCL), n, % | 1 (6.25) |
| Multiple myeloma/plasma cell disorder, n, % | 3 (18.8) |
| **CAR-T cell therapy < 24 months, n, %** | 2 (12.5) |
| DLBCL, n, % | 2 (12.5) |
| **Hematological malignancy on active therapy in last 12 months, n, %** | 8 (50) |
| DLBCL, n, % | 3 (18.8) |
| Chronic lymphocytic leukemia, n, % | 5 (31.3) |
| B cell depleting therapy (any) in last 12 months, n, % | 8 (50) |
| Lymphocyte count x 10^9/L at COVID-19 diagnosis, median (IQR) | 1.2 (0.70 – 1.80) |
| Time from tixagevimab-cilgavimab to COVID-19, days, median, (IQR) | 79 (16 – 135) |
| **Vaccination status** |  |
| ≥ 3 COVID-19 vaccines at time of COVID-19, n, % | 16 (100) |
| ≥ 4 COVID-19 vaccines at time of COVID-19, n, % | 9 (56.3) |
| COVID-19 prior to tixagevimab-cilgavimab, n, % | 3 (18.8) |
| **COVID-19 episode severity** |  |
| Mild, n, % | 14 (87.5) |
| Moderate, n, % | 2 (12.5) |
| **Outpatient antiviral therapy for mild COVID-19** | 13 (81.3) |
| Paxlovid, n, % | 11 (68.8) |
| Outpatient IV remdesivir, n, % | 1 (6.25) |
| Molnupiravir, n, % | 1 (6.25) |
| Supportive care, n, % | 1 (6.25) |
| Time of blood sample pre infection, days, median, (IQR) | 18 (7 – 43) |
| Time of blood sample post infection, days, median, (IQR) | 26 (12 – 41) |
| Reactive antinucleoprotein post infection, n, % | 4 (25) |
|  | **Healthy controls (n = 10)** |
| Sex, male, n, % | 3 (30) |
| Age, years, median, IQR | 29 (25 – 33) |
| 2 dose COVID-19 vaccinated, n, % | 10 (100) |
| Time from last COVID-19 vaccine, days, median, (IQR) | 142.5 (129 – 196) |
| Time of blood sample post infection, days, median, (IQR) | 46 (33.5 – 53) |
| Reactive antinucleoprotein post infection, n, % | 10 (100) |

**Supplementary Table 7. Linear mean regression analysis of ancestral and omicron BA.4/5 RBD IgG post BT for HM patients compared with healthy controls (Figure 3B).**

| **Outcome = Figure 3B Ancestral RBD IgG Post BT** | | **Mean regression** | |
| --- | --- | --- | --- |
| **Factor** | **Category** | **Unadjusted β-coeficient (95% CI) p-value** | **Adjusted β-coeficient (95% CI) p-value** |
| Group | HM | **0.71 (0.18, 1.24) 0.010** | 1.06 (-0.24, 2.37) 0.106 |
|  | Controls | Reference | Reference |
| Age (years) |  | **0.02 (0.00, 0.03) 0.038** | -0.01 (-0.04, 0.03) 0.674 |
| Sex | Male | Reference | Reference |
|  | Female | -0.03 (-0.63, 0.57) 0.916 | 0.13 (-0.43, 0.69) 0.642 |
| Months from BT infection until post BT blood sample |  | -0.05 (-0.45, 0.35) 0.784 | 0.15 (-0.25, 0.54) 0.452 |
|  |  |  |  |
|  |  |  |  |
| **Outcome = Figure 3B Omicron BA45 RBD IgG Post BT** | | **Mean regression** | |
| **Factor** | **Category** | **Unadjusted β-coeficient (95% CI) p-value** | **Adjusted β-coeficient (95% CI) p-value** |
| Group | HM | -0.10 (-0.52, 0.31) 0.609 | -0.33 (-1.37, 0.71) 0.520 |
|  | Controls | Reference | Reference |
| Age (years) |  | -0.00 (-0.01, 0.01) 0.843 | 0.01 (-0.02, 0.03) 0.566 |
| Sex | Male | Reference | Reference |
|  | Female | 0.06 (-0.35, 0.47) 0.770 | 0.04 (-0.41, 0.48) 0.863 |
| Months from BT infection until post BT blood sample |  | 0.06 (-0.21, 0.34) 0.636 | 0.05 (-0.27, 0.37) 0.746 |

| **Tests of normality (p>0.05 = no significant skew, linear mean regression selected)** |  |  |  |  |  |
| --- | --- | --- | --- | --- | --- |
|  |  |  |  |  |  |
| Variable | Obs | W | V | z | Prob>z (test of normality) |
| Figure3B Ancestral | 26 | 0.94992 | 1.432 | 0.736 | 0.23084 |
| Figure3B Omicron | 26 | 0.97508 | 0.713 | -0.694 | 0.75624 |

**Supplementary Table 8. Results of sVNT against Omicron BA.4/5 pre and post breakthrough infection in patients with HM (n=16).**

|  | **Omicron BA.4/5** | | |
| --- | --- | --- | --- |
|  | Pre T-C | Pre infection | Post infection |
| **Total samples** | 16 | 16 | 16 |
| Positive SVNT ≥ 30%, n, % | 13 (81.3) | 13 (81.3) | 13 (81.3) |
| Positive SVNT ≥ 50%, n, % | 12 (75) | 10 (62.5) | 9 (56.3) |
| Negative SVNT < 30%, n, % | 3 (18.8) | 3 (18.8) | 3 (18.8) |

**Supplementary Table 9. Quantile median regression analysis of ancestral and omicron BA.4/5 sVNT post BT for HM patients compared with healthy controls (Figure 3C).**

| **Outcome = Figure 3C Ancestral SVNT Post BT** |  | **Median regression** | |
| --- | --- | --- | --- |
| **Factor** | **Category** | **Unadjusted β-coeficient (95% CI) p-value** | **Adjusted β-coeficient (95% CI) p-value** |
| Group | HM | -0.61 (-1.64, 0.42) 0.231 | -0.11 (-2.09, 1.87) 0.909 |
|  | Controls | Reference | Reference |
| Age (years) |  | -0.01 (-0.04, 0.02) 0.358 | -0.01 (-0.06, 0.04) 0.696 |
| Sex | Male | Reference | Reference |
|  | Female | -0.10 (-1.09, 0.89) 0.837 | 0.13 (-0.72, 0.98) 0.760 |
| Months from BT infection until post BT blood sample |  | 0.09 (-0.58, 0.75) 0.789 | 0.01 (-0.60, 0.61) 0.984 |
|  |  |  |  |
|  |  |  |  |
| **Outcome = Figure 3C Omicron BA45 SVNT Post BT** | | **Median regression** | |
| **Factor** | **Category** | **Unadjusted β-coeficient (95% CI) p-value** | **Adjusted β-coeficient (95% CI) p-value** |
| Group | HM | -26.07 (-58.55, 6.41) 0.111 | -13.04 (-90.85, 64.77) 0.731 |
|  | Controls | Reference | Reference |
| Age (years) |  | -0.55 (-1.35, 0.25) 0.171 | -0.28 (-2.32, 1.77) 0.781 |
| Sex | Male | Reference | Reference |
|  | Female | 1.86 (-35.72, 39.44) 0.919 | 1.00 (-2.32, 1.77) 0.781 |
| Months from BT infection until post BT blood sample |  | 1.86 (-17.22, 20.94) 0.842 | -1.31 (-25.02, 22.40) =.910 |

| **Tests of normality (p<0.05 = significant skew, median regression analysis selected)** |  |  |  |  |  |
| --- | --- | --- | --- | --- | --- |
|  |  |  |  |  |  |
| Variable | Obs | W | V | z | Prob>z (test of normality) |
|  |  |  |  |  |  |
| Figure3C Ancestral | 26 | 0.70561 | 8.418 | 4.366 | 0.00001 |
| Figure3C Omicron | 26 | 0.75664 | 6.959 | 3.976 | 0.00004 |

**Supplementary Table 10. Quantile median regression analysis of percent activated CD4+, CD8+ T cells and T follicular helper cells (Tfh) post BT for HM patients compared with healthy controls (Figure 4A – C).**

| **Outcome = Figure 4A CD4+ T cells Post BT** |  | **Median regression** | |
| --- | --- | --- | --- |
| **Factor** | **Category** | **Unadjusted β-coeficient (95% CI) p-value** | **Adjusted β-coeficient (95% CI) p-value** |
| Group | HM | 0.12 (-0.16, 0.40) 0.384 | 0.21 (-0.26, 0.68) 0.369 |
|  | Controls | Reference | Reference |
| Age (years) |  | 0.00 (-0.00, 0.01) 0.385 | -0.00 (-0.02, 0.01) 0.608 |
| Sex | Male | Reference | Reference |
|  | Female | 0.03 (-0.14, 0.20) 0.711 | -0.01 (-0.21, 0.19) 0.924 |
| Months from BT infection until post BT blood sample |  | -0.04 (-0.11, 0.03) 0.244 | -0.02 (-0.17, 0.12) 0.745 |
|  |  |  |  |
|  |  |  |  |
| **Outcome = Figure 4B CD8+ T cells post BT** |  | **Median regression** | |
| **Factor** | **Category** | **Unadjusted β-coeficient (95% CI) p-value** | **Adjusted β-coeficient (95% CI) p-value** |
| Group | HM | 0.05 (-0.06, 0.16) 0.357 | -0.05 (-0.29, 0.19) 0.672 |
|  | Controls | Reference | Reference |
| Age (years) |  | 0.00 (-0.00, 0.00) 0.609 | 0.00 (-0.00, 0.01) 0.481 |
| Sex | Male | Reference | Reference |
|  | Female | -0.04 (-0.13, 0.05) 0.388 | -0.08 (-0.18, 0.02) 0.122 |
| Months from BT infection until post BT blood sample |  | 0.00 (-0.08, 0.08) 1.000 | -0.01 (-0.08, 0.06) 0.816 |
|  |  |  |  |
|  |  |  |  |
| **Outcome = Figure 4C Tfh cells post BT** |  | **Median regression** | |
| **Factor** | **Category** | **Unadjusted β-coeficient (95% CI) p-value** | **Adjusted β-coeficient (95% CI) p-value** |
| Group | HM | -10.58 (-23.04, 1.88) 0.092 | -16.15 (-47.05, 14.76) 0.290 |
|  | Controls | Reference | Reference |
| Age (years) |  | **-0.30 (-0.56, -0.04) 0.027** | 0.00 (-0.81, 0.81) 0.994 |
| Sex | Male | Reference | Reference |
|  | Female | 10.00 (-2.73, 22.73) 0.118 | 2.03 (-11.27, 15.33) 0.754 |
| Months from BT infection until post BT blood sample |  | -0.41 (-8.13, 7.31) 0.914 | -1.75 (-11.16, 7.67) 0.704 |

| **Tests of normality (p<0.05 = significant skew, median regression analysis selected)** |  |  |  |  |  |
| --- | --- | --- | --- | --- | --- |
|  |  |  |  |  |  |
| Variable | Obs | W | V | z | Prob>z (test of normality) |
| Figure 4A | 26 | 0.63946 | 10.31 | 4.781 | 0.00000 |
| Figure 4B | 26 | 0.69595 | 8.694 | 4.432 | 0.00000 |
| Figure 4C | 26 | 0.80939 | 5.45 | 3.475 | 0.00026 |
