# Supplementary material for: Evolution of Humoral and Cellular Immunity Post–Breakthrough Coronavirus Disease 2019 in Vaccinated Patients With Hematologic Malignancy Receiving Tixagevimab-Cilgavimab
Source: Open Forum Infect Dis. 2023 Nov 2;10(11):ofad550. doi: 10.1093/ofid/ofad550 (PMC10644824; doi:10.1093/ofid/ofad550)
Supplement: ofad550_Supplementary_Data [file ofad550_supplementary_data.zip › Supplementary_Figures_clean.docx]

**Supplementary Figure 1. SARS-CoV-2 ancestral and omicron BA.4/5 anti-RBD IgG titer**

Time points - baseline (V0) before tixagevimab-cilgavimab, 30 days (V1), 90 days (V3), 180 days (V6) post tixagevimab-cilgavimab. Horizontal lines represent median and interquartile range. Statistical analysis applied to compare titer at different time points within the same group (Wilcoxon matched signed ranked pairs) or between two groups (Mann-Whitney U test). Kruskal wallis test was used if 3 or more groups compared.

1. SARS-CoV-2 ancestral and omicron BA.4/5 RBD IgG titer in patients with HM receiving tixagevimab-cilgavimab excluding patients with previous COVID-19 (before V0), n = 14.
2. SARS-CoV-2 ancestral and omicron BA.4/5 RBD IgG titer in patients with HM receiving tixagevimab-cilgavimab as per therapy received for underlying disease, n = 38 for cellular therapy, n = 40 for B cell depleting therapy, n = 15 for other.
3. SARS-CoV-2 ancestral RBD IgG titer in patients with HM receiving tixagevimab-cilgavimab as per underlying disease type with median and interquartile range titer provided in table form, n = 8 for acute leukemia, n = 17 chronic leukemia, n = 26 myeloma, n = 28 aggressive lymphoma, n = 6 indolent lymphoma, n = 8 other.
4. SARS-CoV-2 omicron BA.4/5 RBD IgG titer in patients with HM receiving tixagevimab-cilgavimab as per underlying disease type with median and interquartile range titer provided in table form, n = 8 for acute leukemia, n = 17 chronic leukemia, n = 26 myeloma, n = 28 aggressive lymphoma, n = 6 indolent lymphoma, n = 8 other.

A.

B.

C.

D.

**Supplementary Figure 2. SARS-CoV-2 ancestral and omicron BA.4/5 sVNT**

For all figures: time points - baseline (V0) before tixagevimab-cilgavimab, 30 days (V1), 90 days (V3), 180 days (V6) post tixagevimab-cilgavimab. Horizontal lines represent median and interquartile range. Positive neutralization inhibition percent of ≥ 30% was used as per manufacturer’s instructions, represented by dotted grey line. Statistical analysis applied to compare titer at different time points within the same group (Wilcoxon matched signed ranked pairs) or between two groups (Mann-Whitney U test). Kruskal wallis test was used if 3 or more groups compared. McNemar test used for Figure 2B and 2C to compare proportions as a dichotomous variable between time points.

1. SARS-CoV-2 ancestral and omicron BA.4/5 RBD sVNT in patients with HM receiving tixagevimab-cilgavimab excluding patients with previous COVID-19 (before V0), n = 14.
2. Line graph showing proportion of patients with HM in the overall cohort with positive (≥ 30%) or negative (<30%) omicron BA.4/5 sVNT across the study period.
3. SARS-CoV-2 ancestral and omicron BA.4/5 sVNT in patients with HM receiving tixagevimab-cilgavimab as per therapy received for underlying disease, n = 38 for cellular therapy, n = 40 for B cell depleting therapy, n = 15 for other.
4. SARS-CoV-2 ancestral and omicron BA.4/5 sVNT in patients with HM receiving tixagevimab-cilgavimab as per underlying disease type with median and interquartile range titer provided in table form, n = 8 for acute leukemia, n = 17 chronic leukemia, n = 26 myeloma, n = 28 aggressive lymphoma, n = 6 indolent lymphoma, n = 8 other.

A.

B.

C.

D.

**Supplementary Figure 3. Breakthrough infection cohort**

1. Timing of breakthrough infection (BT) in patients with HM (1-16, y axis) and blood collection sampling time at V0 (baseline), V1 (day 30 post tixagevimab-cilgavimab), V3 (day 90 post T-C), V6 (day 180 post T-C) during study period from calendar days post May 1, 2022 (x axis).
2. Study flow chart of cohort of patients with HM and healthy controls and breakthrough cohort

A.

January 2023

May 1, 2022

November 2022

August 2022

B.

Patients with HM who received tixagevimab-cilgavimab dose 150/150mg n = 93

Healthy controls n = 25

Breakthrough (BT) COVID-19 post tixagevimab-cilgavimab n = 19

COHORT 2

2-dose vaccinated + blood sample post breakthrough COVID-19 n = 10

COHORT 1

3-dose vaccinated + no history of COVID-19 n = 15

Blood samples pre and post

BT infection n = 16

Peptide HLA-tetramers & TAME

assay n = 10

**Supplementary Figure 4. SARS-CoV-2 humoral immune response pre and post breakthrough infection**

Horizontal lines represent median and interquartile range. Statistical analysis applied to compare percent neutralization at different time points within the same group (Wilcoxon matched signed ranked pairs) or between two groups (Mann-Whitney U test). Correlation analysis performed using Spearman’s correlation coefficient.

1. Connecting line graph of SARS-CoV-2 ancestral and omicron BA.4/5 RBD IgG titer prior to tixagevimab-cilgavimab, pre infection and post infection. Red dots (n=3) indicate patients with prior COVID-19 before the study period.
2. Connecting line graph of SARS-CoV-2 ancestral and omicron BA.4/5 sVNT prior to tixagevimab-cilgavimab, pre infection and post infection. Red dots (n=3) indicate patients with prior COVID-19 before the study period.
3. Correlation of anti-RBD IgG titer (omicron BA.4/5) with percent neutralization against omicron BA.4/5 in patients with HM post BT infection.

B.

A.

C.

**Supplementary Figure 5. Cell-mediated immunity pre and post breakthrough infection**

1. Representative FACS plots of CD134^+^CD137^+^CD4^+^ T cells and CD69^+^CD137^+^CD8^+^ by AIM assay pre and post BT infection.
2. Line graph of AIM frequency of CD134+CD137+CD4+ T cells in patients with HM pre and post breakthrough infection in those who did (n=8) or did not (n=8) receive B cell depleting therapy. Mann-Whitney U test used to compare the two groups.
3. Line graph of AIM frequency of CD69+CD137+CD8+ T cells in patients with HM pre and post breakthrough infection in those who did (n=8) or did not (n=8) receive B cell depleting therapy. Mann-Whitney U test used to compare the two groups.
4. AIM frequency of CD134+CD137+CD4+ T cells in patients with HM at baseline time point (V0) pre tixagevimab-cilgavimab who had breakthrough infection (BT) compared to those who did not (No BT) during the study period.
5. AIM frequency of CD69+CD137+CD8+ T cells in patients with HM at baseline time point (V0) pre tixagevimab-cilgavimab who had breakthrough infection (BT) compared to those who did not (No BT) during the study period.
6. Correlation of anti-RBD IgG titer (omicron BA.4/5) with AIM frequency of CD134+CD137+CD4+ T cells and CD69+CD137+CD8+ T cells in patients with HM post breakthrough infection. Spearman’s correlation coefficient used for correlation analysis.
7. Correlation of sVNT (omicron BA.4/5) with AIM frequency of CD134+CD137+CD4+ T cells and CD69+CD137+CD8+ T cells in patients with HM post breakthrough infection. Spearman’s correlation coefficient used for correlation analysis.

A.

P1: Post breakthrough infection

P1: Pre breakthrough infection

SPIKE

DMSO

SPIKE

DMSO


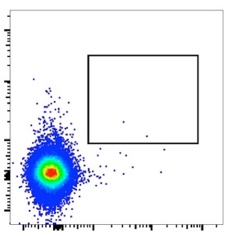

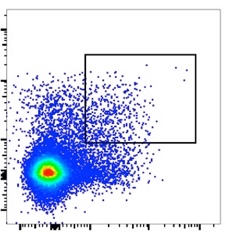

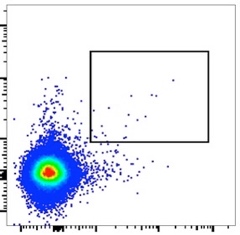

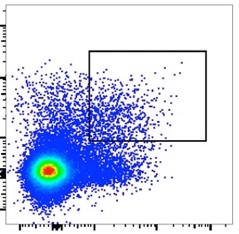


0.18%

0.090%

0.041%

0.022%

0.18%

0.76%

0.003%

0.024%

CD8+ T cells

CD4+ T cells

CD134 (OX40) PE


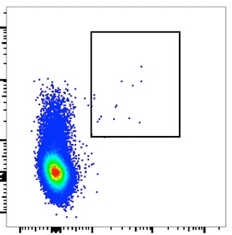

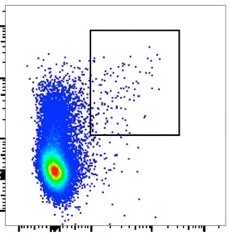

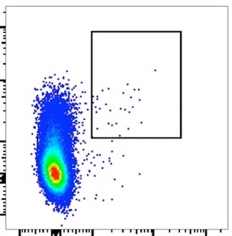

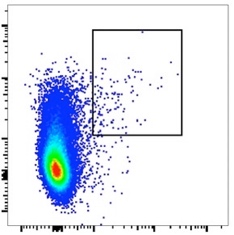


CD69 PerCPCy5.5

CD137 APC

C.

B.

E.

D.

G.

F.


Spearman’s correlation coefficient

CD4+ T cells and omicron BA.4/5 RBD IgG r = 0.19 (p = 0.47)

CD8+ T cells and omicron BA.4/5 RBD IgG r = 0.04 (p = 0.90)

Spearman’s correlation coefficient

CD4+ T cells and omicron BA.4/5 sVNT

r = 0.32 (p = 0.23)

CD8+ T cells and omicron BA.4/5 sVNT

r = 0.10 (p = 0.72)

**Supplementary Figure 6**. **Representative gating strategy for AIM assay**

**
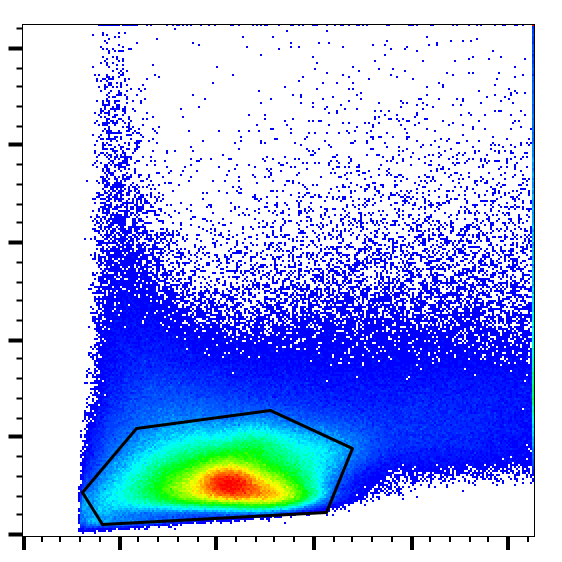

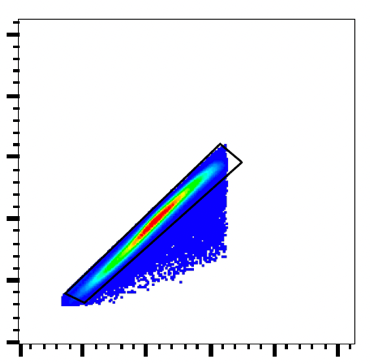

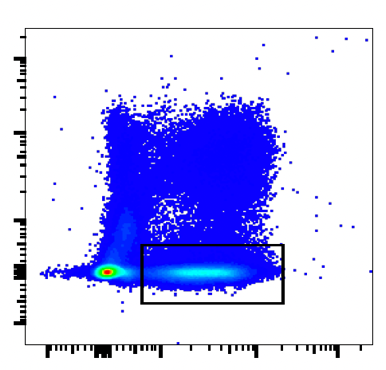
**

CD3 BV510

Live/Dead NIR/

CD14/CD19 APC-H7

FCS-H

SSC-A

FCS-A

FCS-A

**
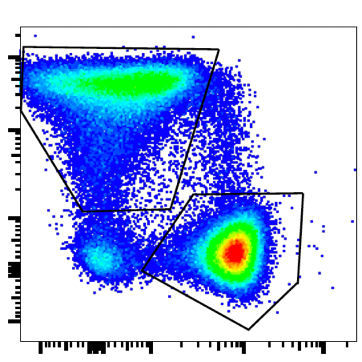

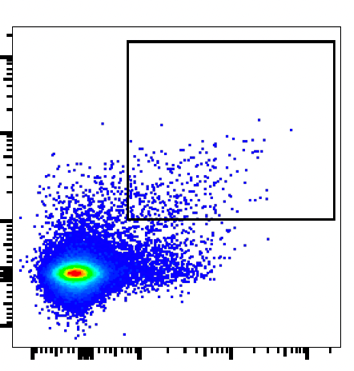

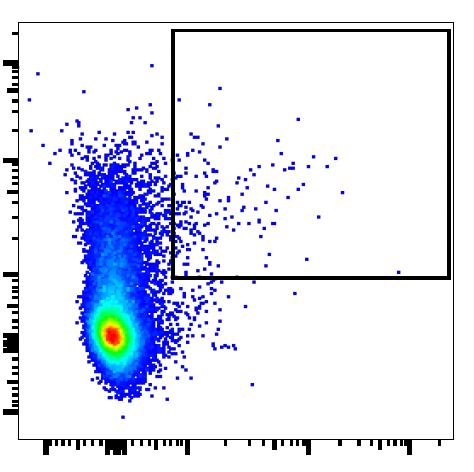
**

CD137 APC

CD69 PerCPCy5.5

CD137 APC

CD134 PE

CD8 BV605

CD4 BV650

**Supplementary Figure 7. Representative gating strategy for TAME assay**

**
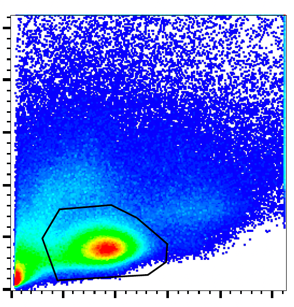

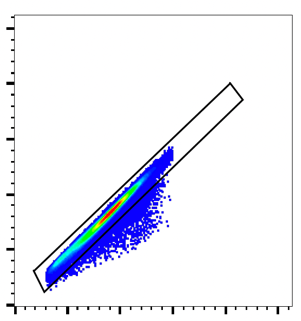

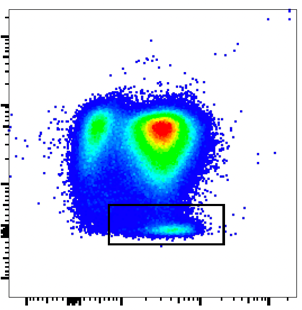

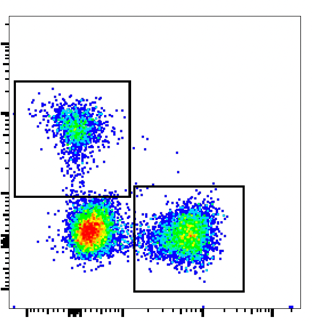
**

Live/Dead NIR/

CD14/CD19 APC-H7

CD4 BV650

CD8 BV605

FCS-H

SSC-A

FCS-A

CD3 BV510

FCS-A

FLOW THROUGH

ENRICHED

UNENRICHED


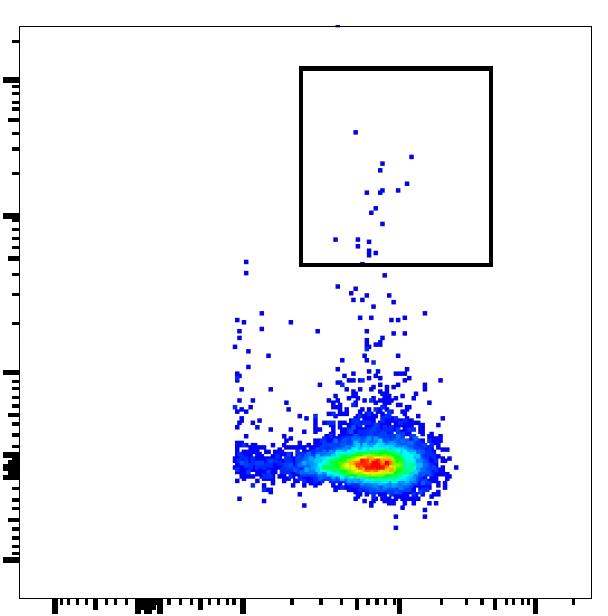

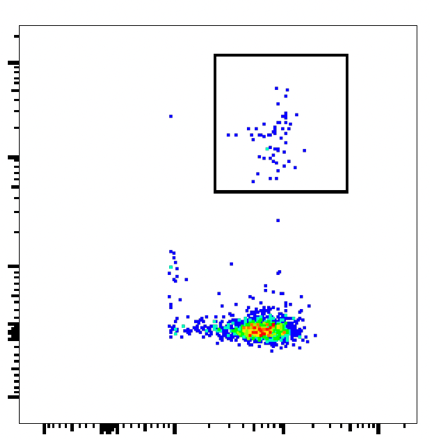

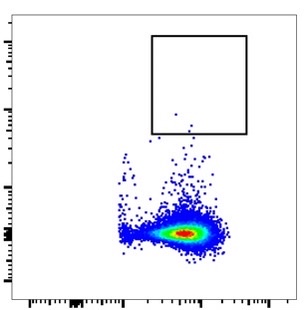


Gated on CD4+ cells

PE

DP4/S_167_


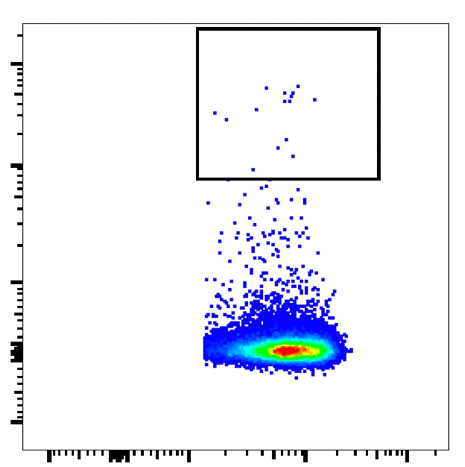

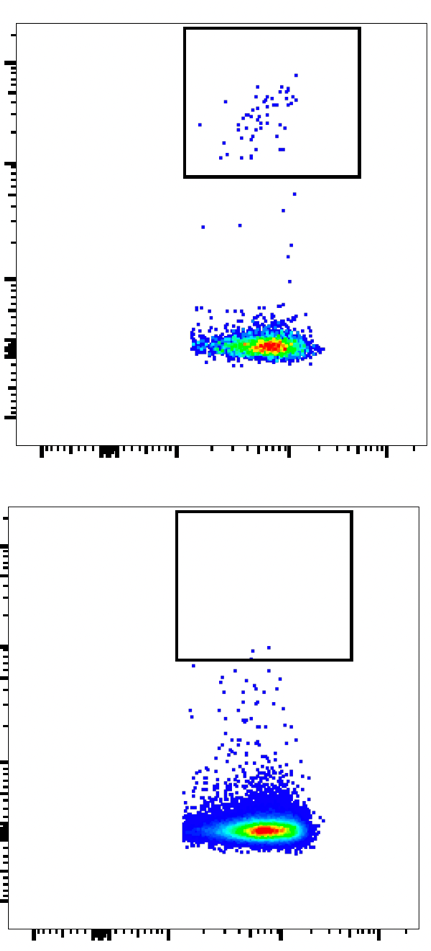

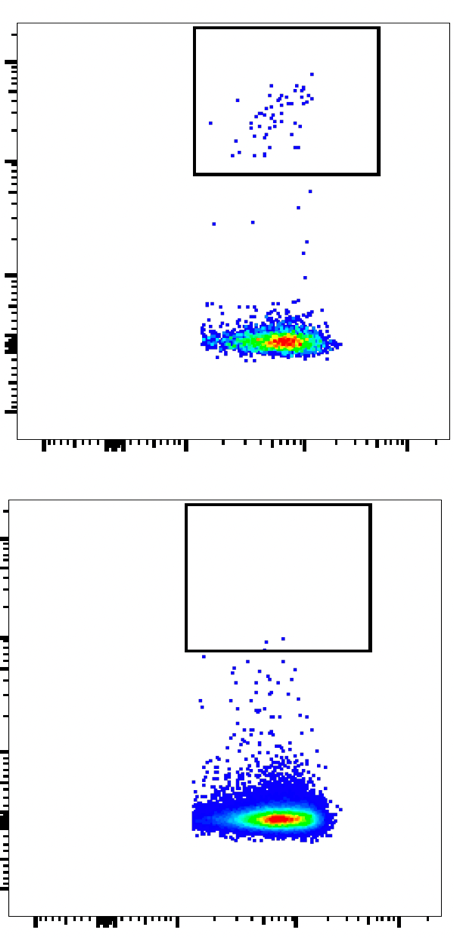


Gated on CD8+ cells

PE

DP4/S_167_

**
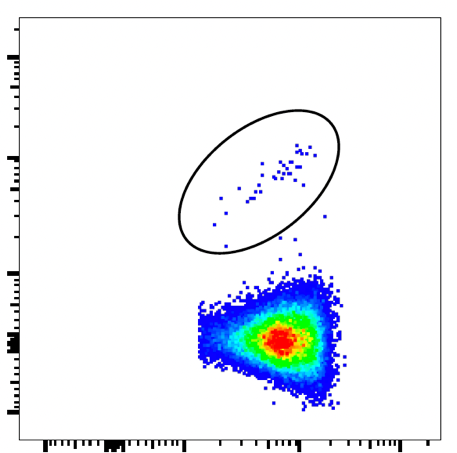

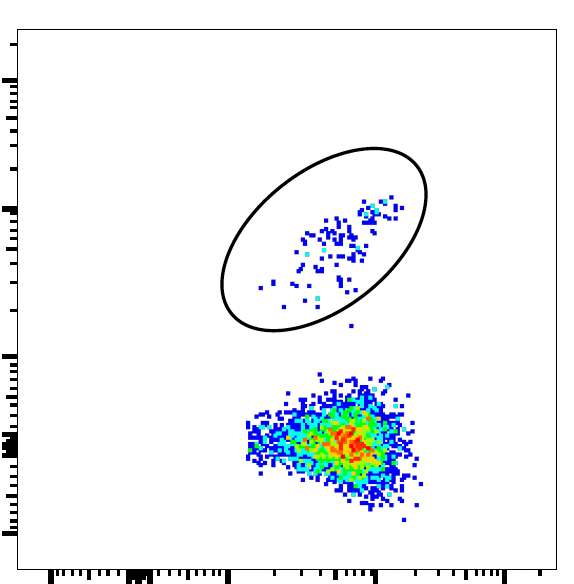

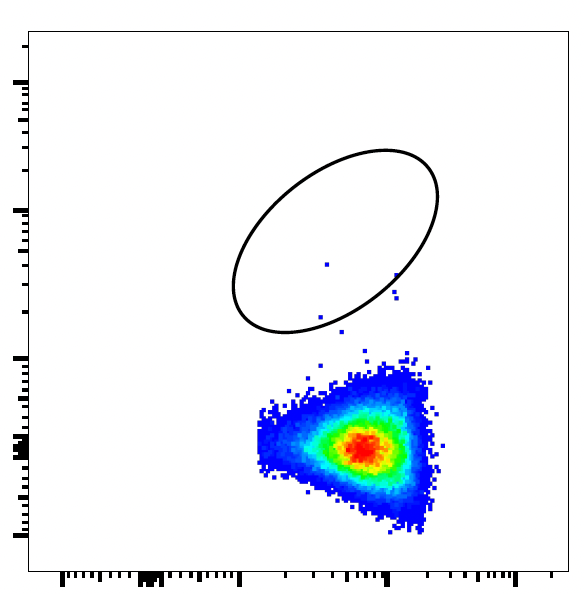
**

APC

B15-S919
